# Phylogeographic analysis delimits three evolutionary significant units of least chipmunks in North America and identifies unique genetic diversity within the imperiled Peñasco population

**DOI:** 10.1002/ece3.7975

**Published:** 2021-07-30

**Authors:** Emily E. Puckett, Sean M. Murphy, Gideon Bradburd

**Affiliations:** ^1^ Department of Biological Sciences University of Memphis Memphis Tennessee USA; ^2^ Wildlife Management Division New Mexico Department of Game & Fish Santa Fe New Mexico USA; ^3^ Department of Integrative Biology Ecology, Evolution, and Behavior Group Michigan State University East Lansing Michigan USA; ^4^ Present address: Department of Forestry and Natural Resources University of Kentucky Lexington Kentucky USA

**Keywords:** climate change, conservation genetics, evolutionary significant units, museum specimens, *Neotamias minimus atristriatus*, phylogeography

## Abstract

Although least chipmunks (*Neotamias minimus*) are a widely distributed North American species of least concern, the southernmost population, *N. m. atristriatus* (Peñasco least chipmunk), is imperiled and a candidate for federal listing as a subspecies. We conducted a phylogeographic analysis across the *N. minimus* range to assess genomic differentiation and distinctiveness of the *N. m. atristriatus* population. Additionally, we leveraged the historical component of sampling to conduct a temporal analysis of *N. minimus* genetic diversity and also considered climate change effects on range persistence probability by projecting a species distribution model into the IPCC5 RCP 2.6 and 8.5 scenarios. We identified three geographically structured groups (West, North, and South) that were supported by both mitochondrial and nuclear data. *N. m. atristriatus* grouped within a unique South subclade but were not reciprocally monophyletic from *N. m. operarius*, and nuclear genome analyses did not separate *N. m. atristriatus*, *N. m. caryi*, and *N. m. operarius*. Thus, while least chipmunks in the Southwest represent an evolutionary significant unit, subspecies distinctions were not supported and listing of the Peñasco population as a Distinct Population Segment of *N. m. operarius* may be warranted. Our results also support consideration of populations with North and West mitogenomes as two additional evolutionary significant units. We found that *N. minimus* genetic diversity declined by ~87% over the last century, and our models predicted substantial future habitat contraction, including the loss of the full contemporary ranges of *N. m. atristriatus*, *N. m. arizonensis*, and *N. m. chuskaensis*.

## Graphical abstract

Least chipmunks are comprised of three evolutionary significant units based on both mitochondrial and nuclear genome analyses. We predicted habitat loss due to climate change, particularly in the southern and western portions of the range, which support active conservation measures for the species.

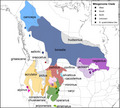

## 1 INTRODUCTION

Accurate taxonomic classification is important for threatened and endangered species to inform resource allocation, population management, and captive breeding efforts (Ryder, 1986; Zachos, 2018). Despite scientific disagreements on both the validity of subspecies and criteria for their delimitation (Haig et al., 2006; Patten, 2015), this taxonomic unit can be protected under the US Endangered Species Act (United States, 1973). Additionally, Distinct Population Segments (DPS) of nonimperiled species and subspecies can also be protected under the ESA, if it can be demonstrated that they are discrete, significant, and, if treated as if they were species or subspecies, their conservation status would meet the criteria for Threatened or Endangered (USFWS & NOAA, 1996). For example, Mexican gray wolves (*Canis lupus baileyi*) are a unique subspecies listed as Endangered under the ESA (National Academies of Sciences, 2019), and the northern population of copperbelly water snakes (*Nerodia erythrogaster neglecta*) is listed as a Threatened DPS under the ESA, even though the status of the southern population is least concern (USFWS, 1997). As such, the ESA provides an effective mechanism for protection and allocation of resources for recovery of threatened and endangered subspecies and populations, even if the species, other subspecies, or other populations are of least concern.

**FIGURE 1:**
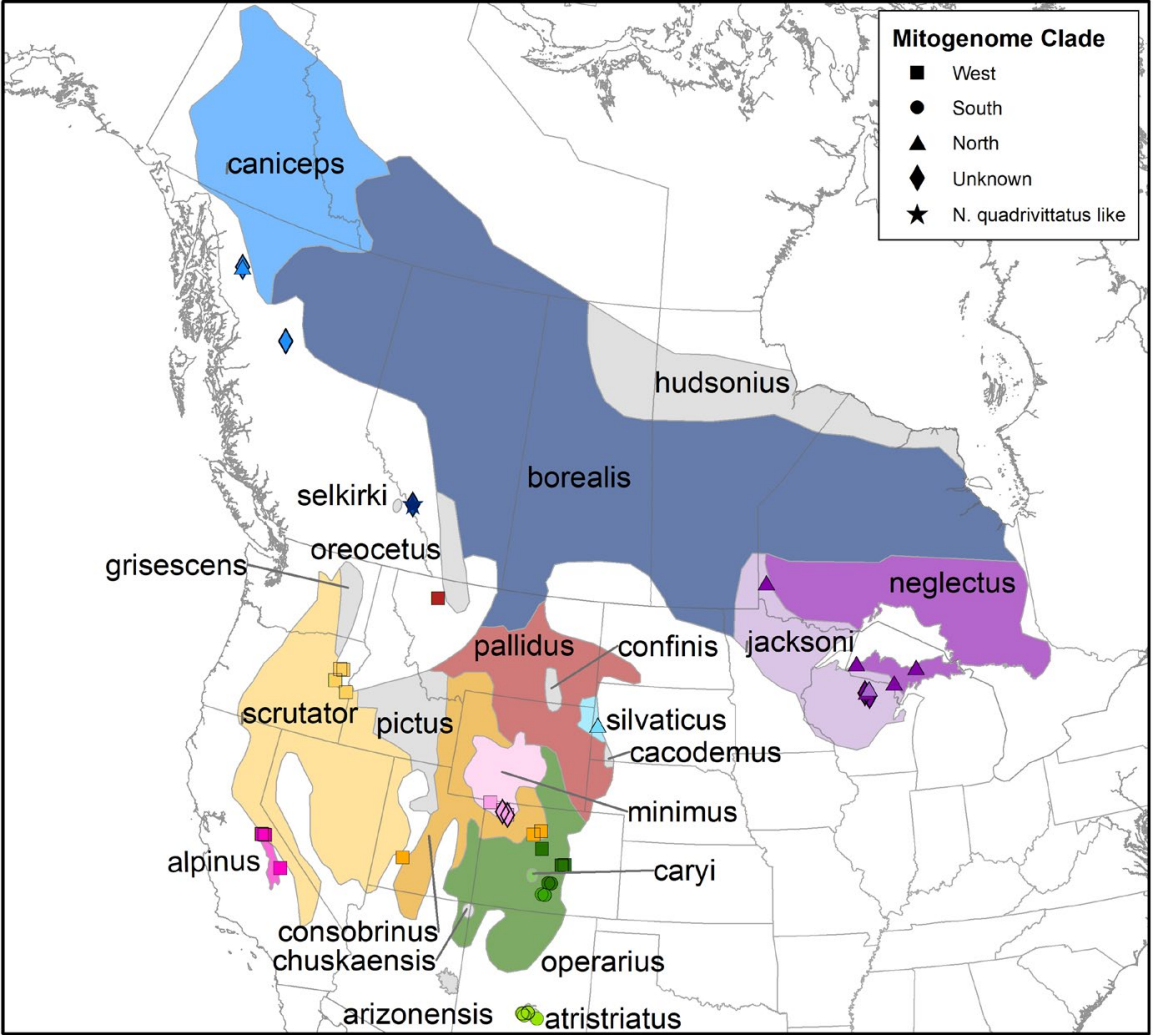
Map of geographic ranges of subspecies boundaries for *N. minimus* and *N. alpinus* (magenta) with sample points overlaid. *N. minimus* subspecies denoted in color if samples obtained from range where subspecies in gray were unsampled; subspecies included *N. m*. *scrutator* (gold), *N. m. consobrinus* (orange), *N. m. minimus* (light pink), *N. m. pallidus* (burgundy; although note samples were closer to *N. m. oreocetus* range), *N. m. operarius* (dark green), *N. m. caryi* (medium green), *N. m. atristriatus* (lime green), *N. m. silvaticus* (light blue), *N. m. caniceps* (medium blue), *N. m. borealis* (indigo), *N. m*. *jacksoni* (light purple), and *N. m. neglectus* (purple). Sample symbol denotes mitochondrial clade, including West (square), South (circle), North (triangle), unknown due to incomplete assembly (diamond), and *N. m. borealis* with *N. quadrivittatus* complex like mitogenome (star). *N. alpinus* and *N. minimus* range maps from IUCN Red List where subspecies boundaries of later were added by georeferencing the map in Verts and Carraway (2001). The IUCN data exclude ranges for *N. m. arizonensis, N. m. atristriatus,* and *N. m*. *selkirki*; therefore, we added those ranges from the USGS (2017) range map

In this study, we set out to evaluate whether the Peñasco population of least chipmunks merits protection under the ESA as a distinct and threatened evolutionary unit of an otherwise widespread and secure species. Least chipmunks (*Neotamias minimus* Bachman, 1839) occur throughout much of North America and are considered of least concern by the IUCN Red List (2019). However, the Peñasco population (*N. m. atristriatus*) in southern New Mexico, USA, is listed as an Endangered subspecies by the State of New Mexico (NMDGF, 2016) and remains a candidate for federal listing under the ESA following a warranted but precluded determination (USFWS, 2012). The only known extant population occurs in the White Mountains of southern New Mexico, following extirpation from the nearby but disjunct Sacramento Mountains by the 1960s (Frey & Hays, 2017). The remaining population is presumably small and faces persistent threats, including habitat loss, anthropogenic conversion of native habitats, drought, wildfire, climate change, and resource competition with gray‐footed chipmunks (*N. canipes*; NMDGF, 2016). Furthermore, the current *N. m. atristriatus* population represents the southernmost range of all least chipmunks (Figure 1). Populations at range edges are often at higher risk of extinction (Wiens, 2016), particularly if these ranges are susceptible to climate change, as is likely the case in southern New Mexico. Climate change has contributed to increasing duration and intensity of droughts, more frequent wildfires, and altitudinal shifts in tree lines on the mountains, thereby reducing the quantity and quality of wildlife habitats (Cahill et al., 2012; Mantyka‐Pringle et al., 2012).

*N. m. atristriatus* were first described in 1913, and thus far, their subspecific uniqueness has been primarily attributed to morphology and habitat. Specifically, they have a larger body size, larger bacular morphology, longer skull, darker pelage and have been captured in different habitat types compared with other proximal least chipmunk subspecies in the Southwest (Bailey, 1913; Conley, 1970; Sullivan & Petersen, 1988). Yet, support for morphological differences was quantitatively nominal in those studies. The finding by Conley (1970) that *N. m. atristriatus* has a longer (by 0.64 mm) occipitonasal skull length than *N. m. arizonensis* had weak statistical support, and five other *N. m. atristriatus* cranial measurements did not differ from other least chipmunk subspecies. In contrast, Sullivan and Petersen (1988) found no differences among *N. m. atristriatus* and all other southwestern least chipmunks for 15 cranial measurements and very weak statistical support for the result that *N. m. atristriatus* had slightly larger overall bacular morphology. Only one genetic analysis has been conducted (Sullivan & Petersen, 1988), but the sample size from the range of *N. m. atristriatus* was two individuals, and the dataset generated to infer evolutionary relationships consisted of allozymes, which have lower power and poorer assignment accuracy compared with other genetic and genomic markers (Allendorf, 2017; Estoup et al., 1998). Consequently, considerable scientific uncertainty surrounds the validity of *N. m. atristriatus* as a subspecies. Moreover, population estimates of genetic differentiation at a regional scale may identify real patterns yet overestimate the importance of such differences when compared to variation at a range‐wide scale. Although least chipmunks have been used as an important empirical test of vicariance in the Southwest following the Last Glacial Maximum (LGM), their range‐wide diversity and differentiation have received less attention.

With the development of new tools, statistical methods, and genomic markers in recent years, the validity of many species and subspecies delimitations for multiple taxa has been scrutinized following range‐wide genetic and genomic analyses (Puckett et al., 2015; vonHoldt et al., 2016). Twenty‐one subspecies have been described across the least chipmunk range (Verts & Carraway, 2001); thus, genetic diversity and differentiation may be substantial. Analyses of *N. m*. *grisescens* suggest that they should be elevated to a unique species because they fall outside of least and alpine (*N. alpinus*) chipmunk diversity in both mitochondrial and nuclear genome analyses (Nordquist, 2015; Reid et al., 2012; Sullivan et al., 2014). Intraspecific variation of the *cytB* mitochondrial gene in *N. minimus* identified two clades with broad geographic distribution, one in the western portion of the range and the second following the Rocky Mountains (Reid et al., 2012). An earlier analysis by Piaggio and Spicer (2000) identified a similar pattern in *N. m*. *scrutator* (in the western range) and a clade split between *N. m. operarius* (in the south) and *N. m. borealis* (in the north). Thus, current mitochondrial data do not support reciprocal monophyly among all named subspecies, and many portions of the range remain unsampled.

Utilizing museum skin samples as a source of genetic material (Ewart et al., 2019), we sought to understand the range‐wide phylogeographic history of least chipmunks and to assess the taxonomic status of the *N. m. atristriatus* population using a larger sample size and a genomic dataset. To explore continuing climatic threats facing *N. m. atristriatus*, we analyzed the decay in heterozygosity over time and also predicted future species distribution into two IPCC5 climate scenarios. The results of our study will aid in the conservation and management of least chipmunks in North America.

## 2 METHODS

We partnered with four museums (Academy of Natural Sciences of Drexel University: ANSP, American Museum of Natural History: AMNH, University of Michigan Museum of Zoology: UMMZ, and the University of Colorado's Museum of Natural History: UCM) to sample prepared specimens from *N. minimus* (*n* = 66; including seven *N. m. atristriatus*), *N. quadrivittatus* (*n* = 16), *N. alpinus* (*n* = 2), and *N*. *umbrinus* (*n* = 3; Figure 1 and Figure S1, Table S1). The date of field collection varied from 1902 to 2009 (Table S1). From each specimen, we cut approximately 9 mm^2^ of dried skin tissue from the ventral side along the seam. We soaked each tissue sample in 1× TE for 24 hr at room temperature (Bi et al., 2013; Burrell et al., 2015) prior to DNA extraction, following the manufacturer's protocol using the Qiagen DNeasy Blood and Tissue Kit with RNase A treatment. We measured DNA concentration using a Qubit 3.0 with the high‐sensitivity assay.

We genotyped samples using an exon probe set (3,617 nuclear loci plus full mitogenome) previously designed for *Neotamias* (see Appendix Text S1 for probe subsetting procedures) (Bi et al., 2012, 2013). DNA was sent to Arbor BioSciences (Ann Arbor, MI) for probe capture and sequencing on one lane of an Illumina HiSeq 4000 (100 bp paired end).

We used the de novo target capture pipeline (Singhal et al., 2015) to process the raw data. Briefly, we removed reads that did not pass Illumina quality filters before trimming reads with trimmomatic v0.38 (Bolger et al., 2014) and then removed adapters with CUTADAPT v1.18 (Martin, 2011). Reads were merged with a modified version of FLASH v1.2.8 (Magoc & Salzberg, 2011; Vieira, 2015), and then, paired reads that overlapped each other were merged with COPE v1.2.5 (Liu et al., 2012). Finally, we mapped reads to an *E. coli* genome using BOWTIE2 (Langmead & Salzberg, 2012) to remove contamination.

We built a pseudo‐reference genome for the eastern chipmunk (*Tamias striatus*) so that we could reference map our reads to an outgroup. We downloaded reads for three eastern chipmunks genotyped using the same exon capture probe set (SRA Accession SRR504642). We first identified the unique barcodes for each individual as the eight base pairs next to the Illumina adapter and then used CUTADAPT to separate reads for each individual. Reads were cleaned with TRIMMOMATIC using default settings and then assembled for each sample into contigs using TRINITY v2.8.4 (Grabherr et al., 2011). We compared contigs to the full exon capture probe sequences using BLAT (Kent, 2002) and then assembled the pseudo‐reference genome by retaining contigs identified as reciprocal matches to the probe set using the make_PGR.py script from the SqCL pipeline (Singhal et al., 2017). We indexed the FASTA output file using BOWTIE2, and then, the cleaned reads from each sample were mapped to the *T. striatus* pseudo‐reference genome using BOWTIE2.

### 2.1 Mitogenome analyses

We assembled a mitochondrial genome for each sample by mapping reads to a *N. quadrivittatus* mitogenome (NCBI Accession KY070142) using the reference‐based short read assembler YASRA (Ratan, 2009). We ran YASRA on both the data collected for this study as well as previously sequenced *N. alpinus* (Table S1). When more than one contig was generated, we imported the data into GENEIOUS PRIME 2019.0.4 (https://www.geneious.com) and reference aligned to the same reference genome to create a single contig. We calculated the amount of missing data in each sample using AMAS (Borowiec, 2016); missing data ranged from 1.9% to 18.7%. We aligned the newly created mitogenomes with previously published *Neotamias* mitogenomes (NCBI accessions KY070142‐KY070197) and *T. striatus* (NCBI SRA: SRR504642) (Bi et al., 2012; Sarver et al., 2017) using the Geneious alignment algorithm in GENEIOUS PRIME.

To understand patterns of differentiation, we first analyzed mitogenomes by building a NeighborNet network in SPLITSTREE v4.14.8 (Huson & Bryant, 2006). We then selected the sequence with the least missing data from each distinct least chipmunk subclade to estimate divergence times. The network analysis revealed three clades for *N. minimus*, where each clade could be divided into two subclades; therefore, we used six *N. minimus* samples. We included two samples of *N. quadrivittatus* (KY070154 and KY070142) and *N*. *umbrinus* (*N*. *u. montanus*: KY070152; *N*. *u. adsitus*: AMNH‐147870 assembled for this study), and one sample each from *N*. *canipes* (NCBI Accession KY070149), *N*. *rufus* (KY070197) and *T. striatus* (NCBI SRA SRR504642) (Sarver et al., 2017). Using the program BEAUTI, we set up a BEAST v2.5.1 (Bouckaert et al., 2014) input file with the following parameters: partitioning of the data into genes and tRNAs, a GTR+Γ substitution model for each partition, linked clock and tree models, a relaxed log‐normal clock (Drummond et al., 2006), and a Yule Model. We specified a log‐normal prior with $\bar{x}$ = 0, *σ* = 0.8, and offset = 7.0 on the root node based upon the estimated divergence time between eastern and western chipmunks (Dalquest et al., 1996). Using the University of Memphis high performance computing cluster (Memphis, TN, USA), we ran 10^8^ MCMC iterations, sampling the chain every 10^4^ iterations after applying a 10% burn‐in (following observation of the posterior trace in TRACER v1.6, where posterior ESS values were >200 for all parameters). We combined two independent chains and then output the tree with the highest median log credibility score using TREE ANNOTATOR v2.5.1; we report the node age and 95% HPD from this tree.

### 2.2 Individual nuclear genome analyses

Following mapping to the *T. striatus* pseudo‐reference genome, we called SNPs in ANGSD v0.920 (Korneliussen et al., 2014) using a minimum quality score of 20, a minimum 60% genotyping rate per locus, and a SNP *p*‐value of 1 × 10^–4^. We estimated Watterson's theta (*θ_w_*) from the called SNPs on the least chipmunk samples in ANGSD.

We further cleaned the data by (in order): applying a per SNP genotyping rate of 20% (‐‐geno in PLINK; Chang et al., 2015; Purcell et al., 2007), removing C to T and G to A sites based upon calls of the ancestral allele (i.e., *T. striatus*) to avoid transitions from postmortem damage, selecting one site per contig (custom R script; Appendix Text S2), and then applying a minimum genotyping rate of 40% to individuals (‐‐mind in PLINK). Given our interest in potentially rare variants, we set MAF >0.01. This resulted in a dataset with 100 individuals and 513 SNPs. To check if samples clustered with other samples of the same species identifier, we ran a PCA (‐‐pca in PLINK) with all five species and a separate analysis only with *N. minimus* and *N. alpinus*. We also ran ADMIXTURE v1.3.0 (Alexander et al., 2009) on all five species with 20 iterations per cluster (*K*: 1–20). We plotted results using the *ggplot2* package in R (Wickham, 2016). We calculated pairwise *F_ST_* in ARELEQUIN v3.5.2.2 (Excoffier & Lischer, 2010); samples were a priori grouped by subspecies designations except for *N. m. operarius* samples, which we split into two groups, each with four samples (see Results). Our ADMIXTURE and *F_ST_* analyses, in conjunction with location data, identified samples that may have been misclassified at either the subspecies or species level (Figures S1 and S2, Table S2). Finally, we tested for isolation‐by‐distance (IBD) by comparing pairwise population *F_ST_* following linearization [*F_ST_*/(1 − *F_ST_*)] and Euclidian distance using the *vegan* package in R (Oksanen et al., 2019).

### 2.3 Phylogenomics

To investigate the tree topology within the nuclear data, we reanalyzed our probe capture data using the full sequences instead of the SNPs. Mapped and assembled loci for each sample were aligned using MAFFT v7.407 (Katoh & Standley, 2013). We used GBLOCKS v0.91 (Castresana, 2000; Talavera & Castresana, 2007) to reduce locus length by removing poorly aligned portions from each locus; specifically, base pairs were retained given the following parameter values: 50% of the sample size was present for conserved positions, 90% of the sample size was present for flank positions, the maximum length of nonconserved positions was 8 bp, and the minimum length of a block after gap cleaning was 12 bp. We used a concatenated approach to infer the tree topology. We retained loci present in 85% of the samples, resulting in 259 loci for a total of 54,051 bp. We concatenated all loci using a custom script in the pipeline (Singhal et al., 2017) and then ran RAxML v8.2.12 (Stamatakis, 2014; Stamatakis et al., 2008) with a *T. striatus* individual designated as the outgroup. We ran 20 iterations of the maximum likelihood algorithm and 1,000 bootstrap replicates.

### 2.4 Modeling diversity over time

To investigate whether least chipmunks experienced a loss of genetic variation through time, as might be expected if anthropogenic habitat loss has driven population declines or increased isolation between demes, we conducted a temporal analysis of genetic diversity, leveraging the historical component of our sampling. We used individuals (*n* = 50) as the unit of analysis rather than grouping samples by location or time period, to avoid introducing potential biases via our clustering scheme. For each sample, we calculated heterozygosity as the proportion of genotyped base pairs at which the individual was called heterozygous divided by the total number of genotyped base pairs in that individual. We then built a novel model, implemented in the Bayesian framework of RStan v2.21.2 (Stan Development Team, 2020) to model individual heterozygosity as a function of sampling year. To account for spatial autocorrelation, we incorporated a spatial covariance into the model. Specifically, we modeled individual heterozygosity as multivariate‐normally (MVN) distributed, with a parametric covariance matrix that was a decaying function of the pairwise geographic distance between individuals; we used a powered exponential function, which is standard in geostatistics (Diggle et al., 1998). The expected heterozygosity for each sample (the vector of means in the MVN) was calculated as the sampling year of each individual multiplied by the estimated slope parameter, plus a global intercept. That is:

$$H_{i}∼MVN\left(\mu_{i}=M+\beta \times T_{i},\;\Sigma =\alpha_{0}\times \exp\left(‐{\left(\alpha_{1}D\right)}^{\alpha_{2}}\right)\right),$$

where *H_i_* is the heterozygosity in the *i*th individual, $\mu_{i}$ is the expected value of the heterozygosity of the *i*th individual, *M* is the global intercept, β is the estimated per‐year effect of time on heterozygosity, $T_{i}$ is the sampling year of the *i*th individual, *D* is the pairwise geographic distance between all individuals, and the α parameters govern the shape of the decay of covariance in heterozygosity between individuals with geographic distance. This model is similar to a linear regression with spatially autocorrelated residuals. To account for possible model misspecification due to both the heteroscedasticity in the data and the fact that our response variable (heterozygosity) is constrained to vary between 0 and 1, while the distribution we used to model it (the multivariate normal) is not, we also analyzed the data using a Poisson regression approach. Because the results were consistent across both modeling approaches, we present only the results of the simpler parameterization in the main text and include the results of the Poisson regression in Appendix Text S3.

To facilitate chain mixing in RStan, we scaled our response and predictor variables. We scaled heterozygosity by dividing all values by the maximum value, sampling year by subtracting the minimum, then dividing all values by the shifted maximum, and geographic distance by dividing by the maximum. All parameter estimates were back‐transformed to account for these scalings and are therefore interpretable within the original scale of the variables. The priors on all parameters were standard normals (i.e., *N*(0,1)), except for $\alpha_{2}$, which was modeled as uniform between 0 and 2, which are the parameter limits over which the powered exponential function is stable. We chose to use only weakly informative priors because we did not have strong beliefs about the parameter values. To explore whether our results were biased by the uneven temporal sampling, and particularly by the small number of modern samples, we dropped all samples collected after 1,952 (*n* = 4) and reanalyzed the remaining samples (*n* = 46) using the same modeling framework described above.

To characterize the posterior probability distribution of this model, we ran four independent chains using the No‐U‐Turn sampling algorithm (NUTS) (Hoffman & Gelman, 2014) for 10^4^ iterations each, discarding warm‐up and recording every 20 iterations to thin the chain. Convergence was assessed by visual inspection of trace plots and marginal parameter posterior distributions and by comparison between independent runs. The significance of the effect of time on heterozygosity was assessed by determining whether the 95% credible interval of the marginal posterior distribution of the parameter contained zero.

### 2.5 Ecological Niche modeling

We downloaded occurrence records for museum specimens of *N. minimus* from VertNet, iDigBio, and Arctos, and then removed duplicates based on combined museum and sample IDs for a total of 15,542 records. Within these records, 12,069 had corresponding geographic coordinates. To model the contemporary distribution, we ran MAXENT v3.3.3k (Phillips et al., 2006). We used all 19 variables in the WorldClim dataset (Hijmans et al., 2005) as well as a layer for altitude; all layers were interpolated on 2.5 arc‐minute grids. We modeled the contemporary distribution of least chipmunks using the 20 data layers and then hindcast the species distribution to the LGM (18–22 kya). For each analysis, we ran 10 replicates (1,000 iterations per replicate), cross‐validation, a regularization multiplier of one, and 10,000 background points with all auto features enabled. Additionally, we projected the species distribution model into two future climate scenarios defined by the IPCC (IPCC, 2014), using the same modeling parameters that were used in the hindcast analysis. The future climate scenarios were the RCM2.6 and RCM8.5 models for climate in 2070 of the 19 climatic variables built with the MIROC‐ESM‐CHEM model (Watanabe et al., 2011). These models are, respectively, associated with a stringent mitigation plan that likely keeps warming below 2℃ in 2,100, and a scenario without efforts to decrease carbon emissions resulting. Thus, these models represent the range of climate change being considered by the IPCC. We converted output into a binary map of predicted presence or absence using the estimated 10% training presence value produced by MaxEnt and averaged across the 10 iterations (logistic threshold range: 0.307–0.850).

## 3 RESULTS

### 3.1 Sample ranges

Several samples in our dataset were obtained from outside the defined subspecies ranges (Figure 1). Specifically, the *N. m. borealis* samples were closer to the *N. m*. *selkirki* range; the *N. m. pallidus* samples were closest to the *N. m. oreocetus* range; the *N. m. caryi* samples were fully within the *N. m. operarius* range; and half of the *N. m. neglectus* samples were within the *N. m*. *jacksoni* range. Although it is fair to question prior researchers' subspecies designations for some of those samples, the available subspecies range maps may have inaccuracies. We digitized the subspecies range map from Verts and Carraway (2001), which may have introduced errors from reprojection, extent errors from the original source (e.g., one sample of *N. m. consobrinus* falls outside the range map), or inaccuracies in the original boundaries. Beyond clustering changes detailed below, we maintain the subspecies names from museum descriptions.

### 3.2 Exon capture metrics

We generated 337 M reads across 87 samples. Data quality varied greatly for each individual, and we removed nine samples because of low data quality based on the average sequencing depth across the target loci (<6×). Average sequencing depth across the remaining 78 samples was 21×. As a check on data quality, we estimated *θ_w_* = 3.7 × 10^–4^ in the *N. minimus* samples. This is within the range estimated for *N. alpinus*, for which the original probe set was developed (Bi et al., 2019). Thus, even though we used a subset of the probes, our data contains similar diversity to a closely related taxon.

### 3.3 Sample clustering

The five species in our analyses showed the best clustering at *K* = 7 based on cross‐validation analysis (Figure S2). This clustering clearly differentiated *N. cinereicollis*, *N. quadrivittatus*, *N*. *umbrinus*, and *N. alpinus*, where the remaining three clusters were identified across the range of *N. minimus*. The ADMIXTURE analysis identified two *N. quadrivittatus* samples that clustered with *N*. *umbrinus* and were sampled in an area of sympatry with *N*. *u. montanus* (Figures S1 and S2). Thus, we grouped these two samples with *N*. *u. montanus* in the phylogenomic analysis.

Four samples labeled as *N. m. operarius* clustered with western samples of *N. minimus* (Figure S2b) and were geographically located on the border of the *N. m. operarius* and *N. m. consobrinus* ranges (Figure 1); the mitochondrial clade in which these four samples were placed was different from that of other *N. m. operarius* samples (see below). Therefore, we separated the eight samples of *N. m. operarius* into two groups for the differentiation analysis and calculated an *F_ST_* = 0.157 (Table S2). Given the moderate *F_ST_* and mitogenome clade, four samples classified as *N. m. operarius* may represent the eastern range of *N. m. consobrinus*.

### 3.4 Mitogenome analyses

**FIGURE 2:**
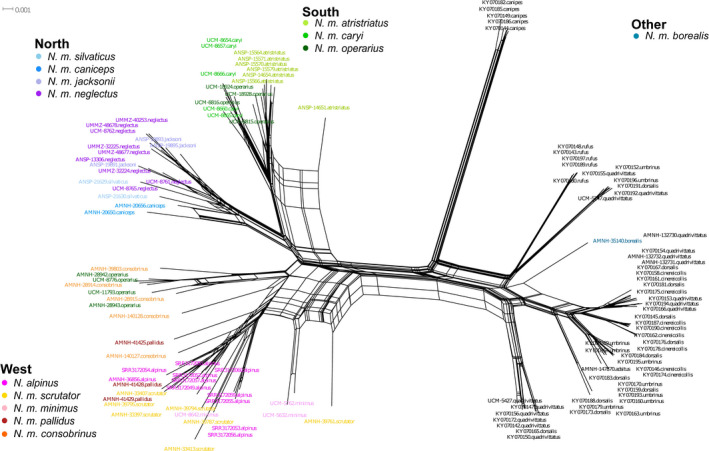
Mitogenome haplotype network of *N. quadrivittatus* species complex (*N. quadrivittatus*, *N*. *umbrinus*, *N. rufus*, *N. cinereicollis*, and *N. dorsalis*—in black), *N. canipes* (black), and *N. minimus* subspecies including *N. alpinus* (see legends for each clade for colors of named subspecies). Note, one sample of *N. m. borealis* clustered near the *N. quadrivittatus* species complex and is shown in medium blue

**FIGURE 3:**
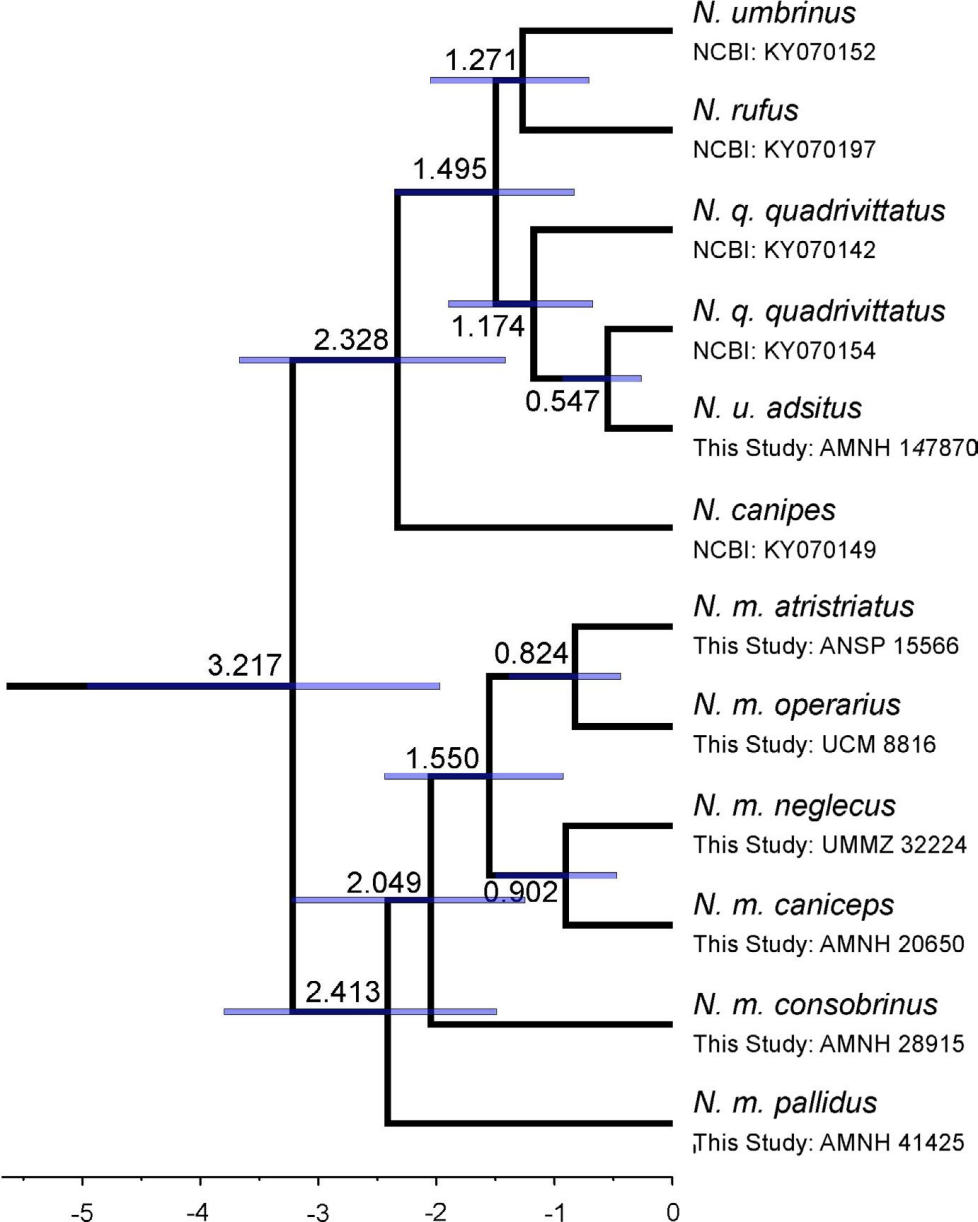
Time‐calibrated phylogenetic tree of *Neotamias* mitogenomes focused on *N. minimus* within‐species diversity. Previously sequenced mitogenomes denoted with NCBI accession number, while mitogenomes produced in this study noted with sample name. Nodes are labeled with the estimated divergence time in millions of years (Mya), and purple bars represent 95% HPD on the divergence time estimate. All Bayesian posteriors on the nodes were 1.00 showing high support for the tree topology. The outgroup, *T. striatus*, was removed from the display

We assembled mitogenomes for 60 samples, which we combined with outgroups from the *N. quadrivittatus* species complex (Table S1) to build a haplotype network. We identified three splits within *N. minimus*: West, South, and North (Figure 2). Each split contained varying amounts of substructure that was concordant with geography (Figure 1). The West contained subspecies in the western and central portions of the range. Within the West, there was an additional split, West‐A, which contained *N. m. consobrinus* and four samples of *N. m. operarius* (see above), and West‐B, which contained *N. m*. *scrutator*, *N. m. consobrinus*, *N. m. minimus*, *N. m. pallidus*, and *N. alpinus* (Figure 2). The South was comprised of samples from the southeastern portion of the range. *N. m. atristriatus* and *N. m. caryi* grouped into South‐A and South‐B, respectively; of the remaining four *N. m. operarius* samples, two each grouped with South‐A and South‐B (Figure 2). The North was comprised of subspecies in the central and northern parts of the range, particularly those that extended across Canada (Figure 2). A split produced the North‐W with all samples of *N. m. silvaticus* and *N. m. caniceps*, whereas North‐E contained *N. m*. *jacksoni* and *N. m. neglectus*. The one assembled mitogenome from a sample of *N. m. borealis* (expected to fall within the north clade based on geography) clustered with the *N. quadrivittatus* species complex (Figure 2). Using a representative sample from each subsplit, we estimated the timing of the last common ancestor of *N. minimus* to be 2.41 Mya (95% HPD: 1.49–3.80 Mya; Figure 3). Divergence between South‐A and South‐B was approximately 824 kya (95% HPD: 440–1,385 kya).

### 3.5 Nuclear analyses

**FIGURE 4:**
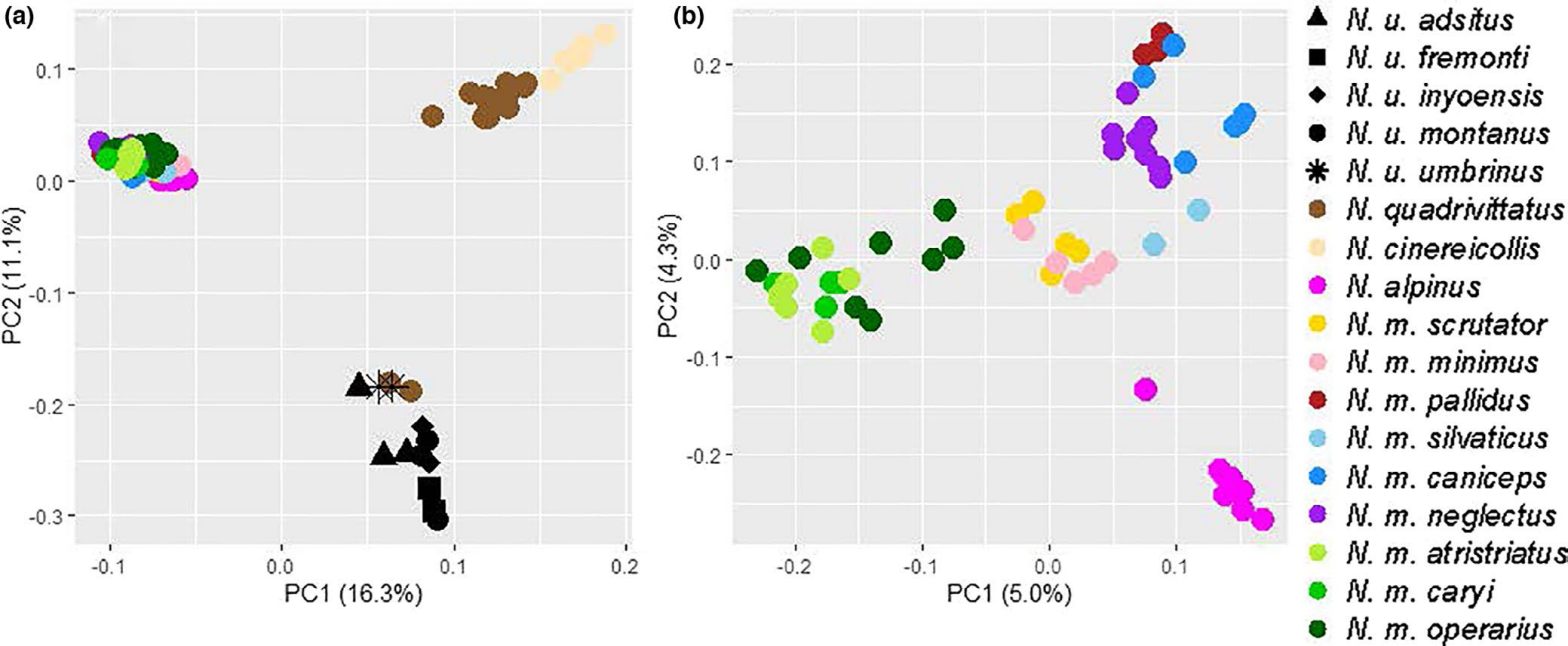
Principal component analysis of genetic variation (513 SNPs) for five *Neotamias* species. (a) PC axes 1 and 2 differentiate the species consistent with phylogenetic analyses. (b) PCA of *N. minimus* and *N. alpinus* data highlights differentiation within *N. minimus* on axis 1, while differentiation between the species on axis 2

**FIGURE 5:**
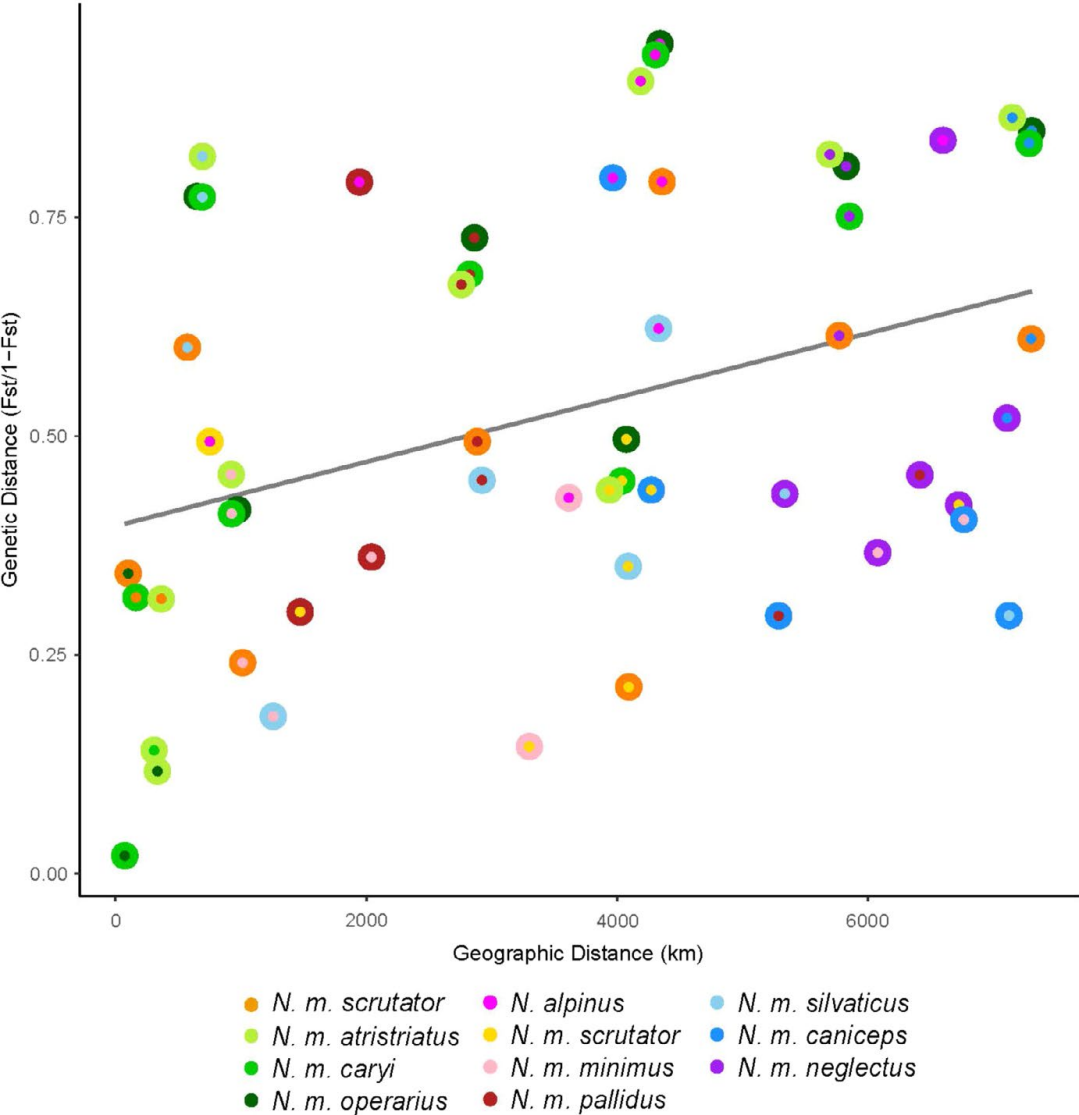
Isolation‐by‐distance (IBD) plot for all pairwise populations of *N. minimus* and *N. alpinus* using 513 SNPs was significant (Mantel's *r* = 0.363; *p* = .022). Each concentric circle represents the comparison between two populations denoted by the colors. The gray line indicates the predicted relationship between pairwise genetic and geographic distances

The first and second axes of our PCA using all species distinguished *Neotamias* species and accounted for 16.3% and 11.1% of the variation in the data, respectively (Figure 4a). *N. alpinus* clustered with *N. minimus* on these two axes, yet separated from least chipmunks on the second axis (4.3% of the variance; Figure 4b) of the ingroup PCA. The first axis of the ingroup PCA separated *N. minimus* samples (5.0% of the variance), distinguished the southern (*N. m. atristriatus, N. m. caryi,* and *N. m. operarius*), western/central (*N. m*. *scrutator* and *N. m. minimus*), and northern (*N. m. pallidus, T. m silvitacus, N. m. caniceps,* and *N. m. neglectus*) clusters of least chipmunks (Figure 4b). The third PC contained 2.9% of the variation and separated samples across the northern cluster. IBD was significant across the range (Mantel's *r* = 0.363; *p* = .022; Figure 5) but suggested that *N. m. atristriatus* had less differentiation from *N. m. operarius* than expected based on distance.

**FIGURE 6:**
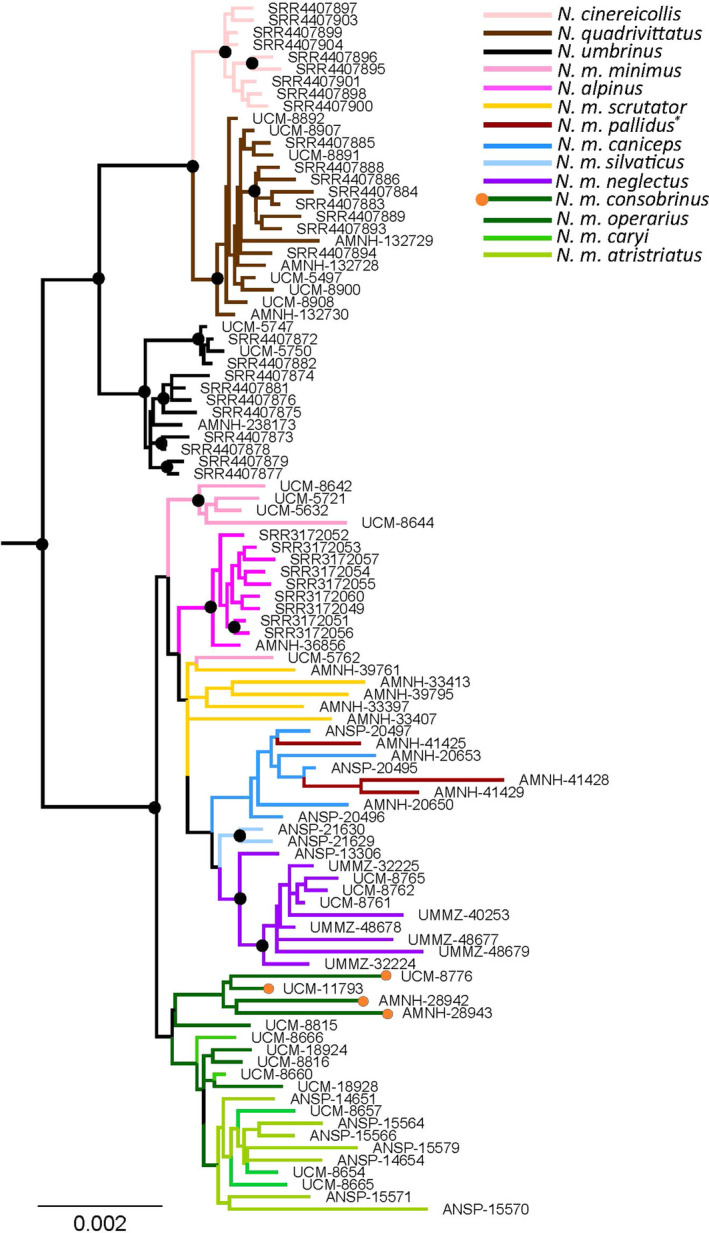
Concatenated phylogenomic tree of *Neotamias* samples (*N. cinereicollis* [peach], *N. quadrivittatus* [dark brown], *N. umbrinus* [black], *N. alpinus* [magenta], and *N. minimus* (*N. m*. *scrutator* (gold), *N. m. minimus* (light pink), *N. m. pallidus* (burgundy, although note samples were closer to *N. m. oreocetus* range), *N. m. operarius* (dark green; where samples that cluster with *N. m. consobrinus* appear with orange dots at tips), *N. m. caryi* (medium green), *N. m. atristriatus* (lime green), *N. m. silvaticus* (light blue), *N. m. caniceps* (medium blue), *N. m*. and *N. m. neglectus* (purple)) with the *T. striatus* outgroup removed for readability. Black circles on nodes indicate bootstrap support greater than 75%

We retained 259 contigs for which more than 85% of samples had an assembled sequence and then built a phylogeny in RAxML from the concatenated alignment. There was strong bootstrap support for the differentiation between the southern populations (*N. m. operarius*, *N. m. caryi*, and *N. m. atristriatus*) along with *N. m. consobrinus* and the western and northern clusters (Figure 6). There was weak support for the backbone topology within the southern clade, indicating little substructure within these samples. The split between *N. m. minimus* and the remaining western and northern clusters was weakly supported. Populations with North mitogenomes clustered together (Figure 6), and samples of *N. m. pallidus* fell within the clade of *N. m. caniceps*.

### 3.6 Diversity over time

**FIGURE 7:**
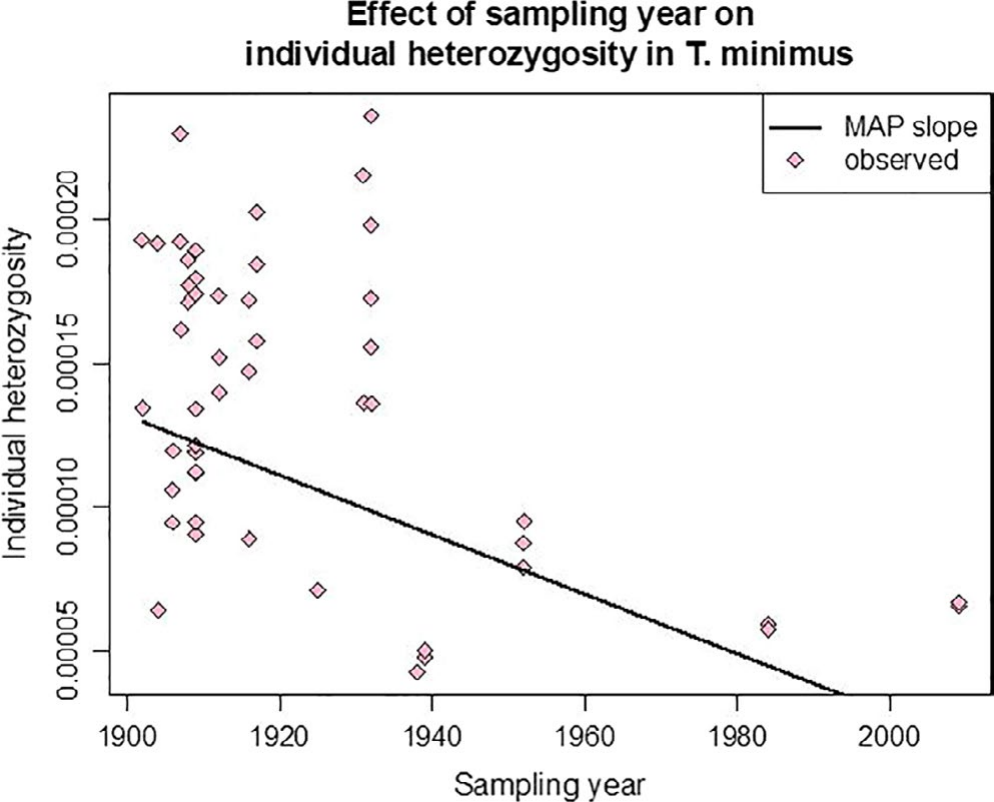
Individual heterozygosity of *Neotamias minimus* (*n* = 50, pink) with rate of decay over time (black line) estimated while controlling for spatial autocorrelation

After controlling for spatial nonindependence between samples, we found that individual heterozygosity in *N. minimus* samples experienced a statistically significant decrease through time. The mean estimated effect size of year in our model was −1.05 × 10^–6^ (95% credible interval (CI): −1.56 × 10^–6^ to −5.55 × 10^–7^; Figure 7), indicating an average reduction of individual heterozygosity by 1.05 × 10^–6^ per year. Over the 107‐year sampling period, this corresponds to a model‐predicted 87% reduction in individual heterozygosity. The finding that individual heterozygosity has decayed over time was strongly supported (Figure S3) and consistent across independent runs. This result was robust to the exclusion of samples collected after 1952; the estimated decay of heterozygosity with year was, in fact, stronger for the dataset consisting solely of pre‐1952 individuals (mean = −1.77 × 10^–6^; 95% CI: −2.85 × 10^–6^ to −7.11 × 10^–7^). We also found a statistically significant decay of heterozygosity using a parallel modeling approach (Poisson regression, described in Appendix Text S3) that accounted for heteroscedasticity and heterogeneity in the number of genotyped SNPs across samples.

### 3.7 Ecological Niche modeling

**FIGURE 8:**
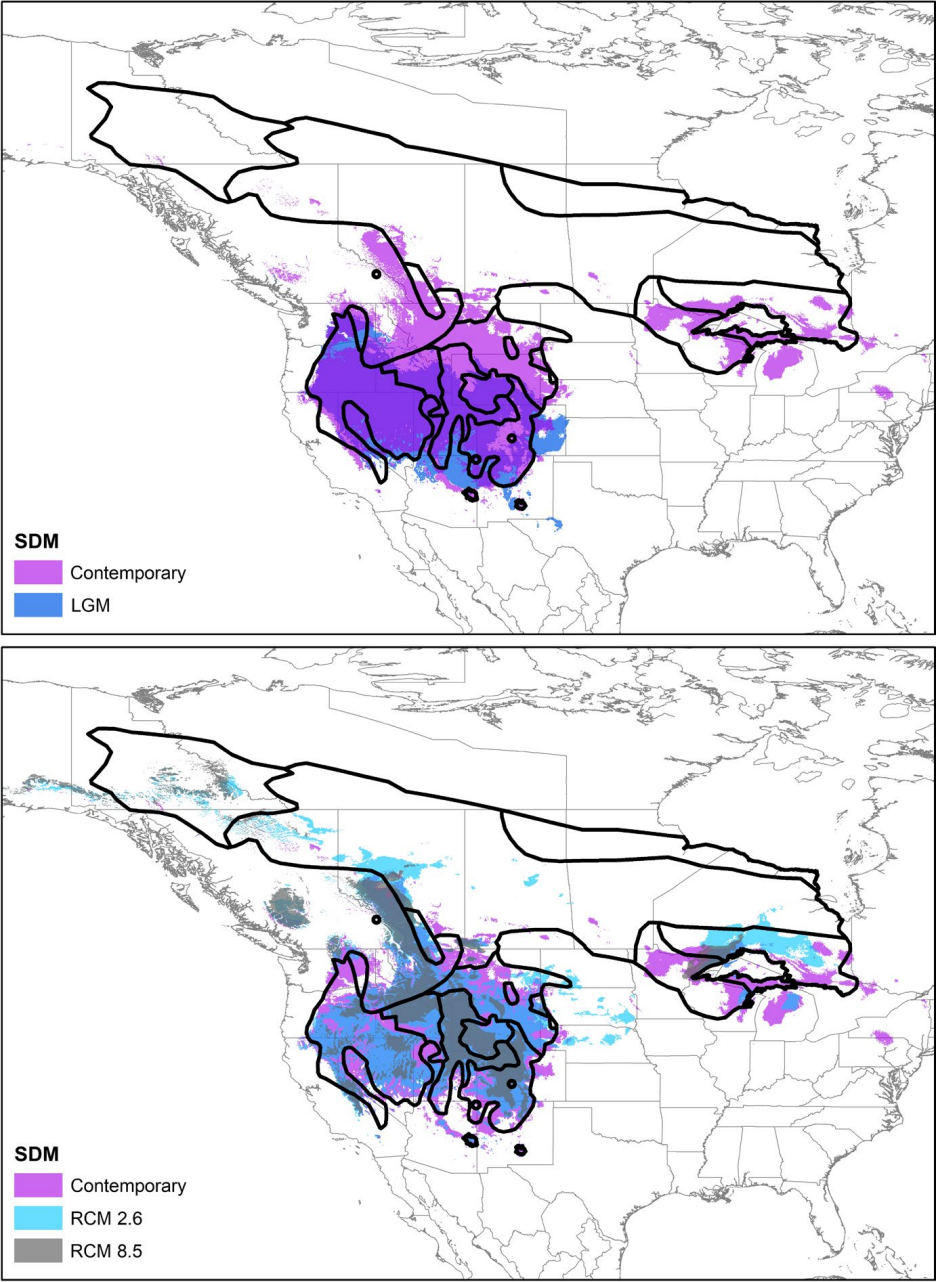
*N. minimus* species distribution maps (SDM) for current, hindcast, and forecast models with subspecies range map overlaid. (Top) Map of contemporary (purple) and Last Glacial Maximum (blue) hindcast overlaid, where occurrence was determined based upon the 10% training threshold cutoff within MaxEnt. (Bottom) Overlaid maps of contemporary (purple), RCM 2.6 (turquoise), and RCM 8.5 (dark gray) forecast species distributions using the same occurrence threshold

Our MAXENT models had high discriminative power, with an average area under the receiver operating curve of 0.871. We used the logistic threshold associated with the 10% training presence (0.306) as the cutoff for defining areas of predicted presence (Figure 8a). The model performed poorly in the northern portion of the range, which also had sparser occurrence records. The hindcast to the LGM estimated that the range has been stable from Washington to California in the west and expanding across the Great Basin into the southern Rocky Mountains (Figure 8a). The disjunct range of *N. m. atristriatus* appears to have been stable since the LGM.

We forecast the contemporary distribution of least chipmunks into both the RCM 2.6 and RCM 8.5 climate models (Figure 8b). Across the range, the predicted distribution either shrank, expanded northwards, or disappeared. For *N. m. atristriatus*, no habitat was predicted under either the RCM 2.6 or 8.5 models (Figure 8b). Similarly, the ranges for *N. m. chuskaensis* and *N. m. grisecens* were not predicted to be occupied under either model and the *N. m. arizonensis* range was not predicted under the RCM 8.5 model. The core of the least chipmunk range (encompassing *N. m. pictus, N. m. consobrinus, N. m. minimus,* and *N. m. operarius*) appeared stable within both climate scenarios and extended westward and eastward in the RCM 2.6 model. The forecast models predicted westward expansion of the range near the current boundaries of *N. m. oreocetus* and *N. m. borealis*. In the eastern portion of the range, the RCM 2.6 model predicted an expansion of habitat northward of Lake Superior; however, that patch was greatly reduced under the RCM 8.5 model (Figure 8b).

We limited our interpretation of these results to the western and southern clades because the contemporary distribution model predicted low probabilities of occurrence across the northern range (Figure 8a). Specifically, the model did not capture the extent of *N. m. hudsonius*, *N. m. borealis*, or *N. m. caniceps*, likely because of limited or absent geographic locations in the databases used for training. This poor model performance may bias estimates of occurrence in the hindcast and forecast analyses. We attempted to address this poor performance by modeling only the northern range; this analysis predicted a larger suitable area in the contemporary climate but an unrealistic hindcast that suggested range stability across the north despite the fact that glaciers covered the study area (data not shown).

## 4 DISCUSSION

To understand the level of genomic distinctness of the imperiled Peñasco least chipmunk, *N. m. atristriatus*, we inferred the phylogeographic history of least chipmunks across North America. *N. m. atristriatus* were previously delimited as a subspecies based upon quantitatively nominal and weakly supported morphological differences as well as limited allozyme data from regional studies of southwestern least chipmunks (Sullivan & Petersen, 1988). Using the definition for evolutionary significant unit (ESU) from Fraser and Bernatchez (2001) of a within‐species lineage that has highly restricted gene flow with other lineages, our range‐wide analyses suggest the delimitation of three ESUs within least chipmunks, one each in the western, southern, and northern portions of the geographic range (Figure 2, Figure S2). Within the southern clade, our genetic data do not support the current distinct subspecies designation for *N. m. atristriatus*.

Populations of *N. m. atristriatus* and *N. m. operarius* had the lowest pairwise *F*_ST_ values (0.094–0.128) compared with other pairwise populations across the range (0.161–0.426; Table S2, Figure 5). These *F*_ST_ values still represent moderate differentiation between *N. m. atristriatus* and *N. m. operarius*, which is likely a consequence of genetic drift caused by geographic isolation of the *N. m. atristriatus* population from other populations. Although *N. m. atristriatus* contains unique mitochondrial diversity that we estimate diverged from its most recent common ancestor ~824 kya, the clustering analyses, nuclear phylogenomic tree, and mitogenome haplotype network unequivocally grouped *N. m. atristriatus* with *N. m. operarius* and *N. m. caryi* in the southern clade. This strongly suggests that these three subspecies may warrant taxonomic reduction to a single subspecies, *N. m. operarius*, which has seniority due to earliest description. Similarly, taxonomic revision should also be considered for the northern and western clades as they also represent ESUs composed of multiple currently defined subspecies.

Considering the conflict between the results of our genetic analyses and previous morphological studies (Conley, 1970; Sullivan & Petersen, 1988) in the context of the diagnosability version of the (sub)species concept (Taylor et al., 2017), it is unclear what diagnosable, heritable character could be used to correctly determine that a least chipmunk specimen of *unknown origin* was *N. m. atristriatus*. Indeed, both Conley (1970) and Sullivan and Petersen (1988) posited that the geographical origin of a specimen was perhaps the most useful attribute for identifying *N. m. atristriatus*. Notably, none of the *N. m. atristriatus* samples grouped with the morphologically similar and sympatric *N*. *canipes* in our mitogenome network, indicating that the *N. m. atristriatus* specimens from which our samples were obtained were unlikely to have been misclassified when collected and archived (Frey & Hays, 2017). An important consideration for a diagnosable characteristic is that it would not require euthanasia of an imperiled species; thus, a blood draw and sequencing a mitochondrial gene would allow for persistence of the individual.

The proposed taxonomic reduction should not necessarily thwart protection of the existing *N. m. atristriatus* population under the ESA, because the population may constitute a distinct population segment (DPS) of *N. m. operarius*. The ESA uses three criteria to define a DPS: Discreteness, significance, and conservation status (USFWS & NOAA, 1996). The Peñasco population is discrete based on its geographic location, which is disjunct and isolated from all other least chipmunk populations. Although the population is not monophyletic relative to other least chipmunk populations, Peñasco individuals have unique mitochondrial diversity and exhibit moderate genetic differentiation from *N. m. operarius* (Table S2), lending support to the significance criterion. Finally, the population has a priority score of 6, indicating a high but not imminent threat magnitude for extinction (USFWS, 2012). However, our RCM 2.6 forecast model predicted extirpation of *N. m. atristriatus* range by 2070 under a model of less than2℃ increase in global temperature (Figure 8b), suggesting the threat of extinction may in fact be imminent. Considering the federal‐level evaluation and the enduring categorization by the State of New Mexico as Endangered, the *N. m. atristriatus* population also meets the conservation status criterion for listing as a DPS.

Our results also have broader implications for least chipmunk conservation across North America. We estimated that heterozygosity has declined by 87% in *N. minimus* over the last century (Figure 7). This result was concordant with an analysis that quantified within‐population heterozygosity decline of 5.4% across vertebrate and invertebrate taxa since the mid‐1800s (Leigh et al., 2019), although our statistical approach differed from that study. However, multiple caveats exist for the interpretation of our heterozygosity loss results. First, because the history of and connectivity among the sampled areas are too poorly understood, we did not directly model the demographic history of the samples and instead applied a phenomenological model. Although such an approach might be more robust, the deterministic functions we applied have no biological basis, which limits the generalizability of our results. Second, there was a temporal signal in the average sequencing coverage across samples (higher coverage in more recent samples); however, we would expect that decreased coverage should lead to an underestimate in heterozygosity (as the SNP caller is less likely to call a heterozygous genotype from fewer reads), so our results should be robust to the effect of coverage. Finally, it is possible that the higher heterozygosity in the older samples is a spurious result due to postmortem DNA damage (Bi et al., 2013; Stiller et al., 2006). Although we took standard bioinformatic steps to mitigate that signal, we cannot rule out the possibility that they were insufficient, so these results should be interpreted cautiously.

Nevertheless, maintaining genetic diversity is important for population persistence and adaptation, particularly to changes or perturbations in the environment. Beyond demonstrating that genetic diversity losses have already taken place, our forecast model predicted extirpation of *N. m. atristriatus, N. m. arizonensis*, *N. m. chuskaensis*, and *N. m*. *grisescens* along with range contractions of *N. m. operarius*, *N. m*. *scrutator*, and *N. m. confinus*. This suggests that habitat conservation and population monitoring may need to occur in many range edge populations.

### 4.1 Least chipmunk phylogeography

The relationships recovered among the major geographic groups using mitochondrial and nuclear data were discordant across the least chipmunk range (Figures 2, 3, and 5), which has also been observed at the species level in *Neotamias* (Reid et al., 2012; Sullivan et al., 2014). The earliest fossils are from northern Colorado (0.30–1.80 Mya; Barnosky and Rasmussen (1988)), which comprises the eastern edge of the modern West ESU. Our mitogenome tree suggests that the South and North groups expanded from an ancestral West population (Figure 3). A westward range expansion from the ancestral range was also consistent with the nuclear data, analyses of which suggested that the *N. alpinus* and *N. m*. *scrutator* populations descended from the centrally located *N. m. minimus*. Previous work estimated *N. alpinus* and *N. m*. *scrutator* diverged from each other at least 446 kya (Rubidge et al., 2014); thus, it is likely that the initial westward range expansion began before that time.

Our analyses provide additional context regarding the substructure and range expansion timing of the North ESU. We estimated that the North‐W and North‐E subclades diverged approximately 902 kya (95% HPD: 472–1,500 kya). While taxonomic identity of fossil fragments of small rodents should be thoughtfully considered, *N. minimus* fossils were identified near the western range edge in Yukon, CA, and dated to 190–780 kya (Storer, 2004), suggesting eastern Beringia as a possible glacial refugium for northwestern populations; a southwards recolonization of this population provides a possible explanation for the sister relationship between *N. m. pallidus* and *N. m. caniceps*. In the east, *N. m. neglectus* may have moved south of the ice during the LGM, which would explain the presence of *N. minimus* fossils as far south as Virginia, USA, dated within the last 0–100 kya (Grady, 1984; Guilday et al., 1977). Our working model suggests that extensive sampling across the northern range may identify one or more secondary contact zones.

Both mitochondrial and nuclear data supported the clustering of the southern range populations (despite mito‐nuclear discordance), although some gene flow with *N. m. consobrinus* was inferred (Figures 4 and 6, Figure S2). Interest in southwestern least chipmunks has been high due to tests of the vicariance hypothesis since the LGM. We estimated the South subclades diverged 824 kya (95% HPD: 440–1,385 kya). Our hindcast model, which predicted unsuitable habitat since the LGM between the Sandia and Sacramento Mountains, offered further evidence against a post‐LGM founding (Figure 8a). Although we inferred that least chipmunks did expand southward from the ancestral range (Figure 6), this likely occurred further in the past than previously hypothesized (Sullivan, 1985). Thus, across the species' range, we did not observe that least chipmunk phylogeography was strongly structured during the LGM.

## 5 CONCLUSIONS

We used genomic data to test if subspecific status of *N. m. atristriatus*, an imperiled population of least chipmunk, was supported when compared to range‐wide patterns of divergence. Neither mitochondrial nor nuclear datasets identified reciprocally monophyletic diversity between *N. m. atristriatus* and the geographically proximate *N. m. operarius* and *N. m. caryi*. We suggest taxonomic reduction of these three subspecies into a single taxon representative of the southern evolutionary significant unit in least chipmunks. Investigation of other southern populations, particularly *N. m. arizonensis* and *N. m. chuskanensis*, may support their inclusion into this ESU as well. Additional subspecies revisions may be warranted across the range of least chipmunks, as our data also support delimiting northern and western ESUs. Finally, predicted geographic occurrence patterns under two climate forecasts suggest loss of habitat across the southern range. This information should be useful for managers and conservationists working to conserve least chipmunks across North America.

## CONFLICT OF INTEREST

Author SMM was an employee of the grant administering agency, New Mexico Department of Game and Fish. Authors EEP and GB declare no conflicts of interest.

## AUTHOR CONTRIBUTIONS

**Emily E. Puckett:** Conceptualization (supporting); data curation (lead); formal analysis (lead); funding acquisition (equal); methodology (lead); project administration (lead); visualization (lead); writing‐original draft (lead); writing‐review & editing (equal). **Sean M. Murphy:** Conceptualization (lead); data curation (supporting); funding acquisition (equal); writing‐original draft (supporting); writing‐review & editing (equal). **Gideon Bradburd:** Formal analysis (supporting); methodology (supporting); visualization (supporting); writing‐review & editing (equal).

## Supporting information

Appendix S1

## Data Availability

DNA sequence data from probe captures have been deposited in the NCBI SRA under BioProject PRJNA734912.
