# Supplementary material for: Phylogeographic analysis delimits three evolutionary significant units of least chipmunks in North America and identifies unique genetic diversity within the imperiled Peñasco population
Source: Ecol Evol. 2021 Jul 30;11(17):12114–28. doi: 10.1002/ece3.7975 (PMC8427584; doi:10.1002/ece3.7975)
Supplement: Supplementary file 1 — Appendix S1 [file ECE3-11-12114-s001.docx]

**APPENDIX**


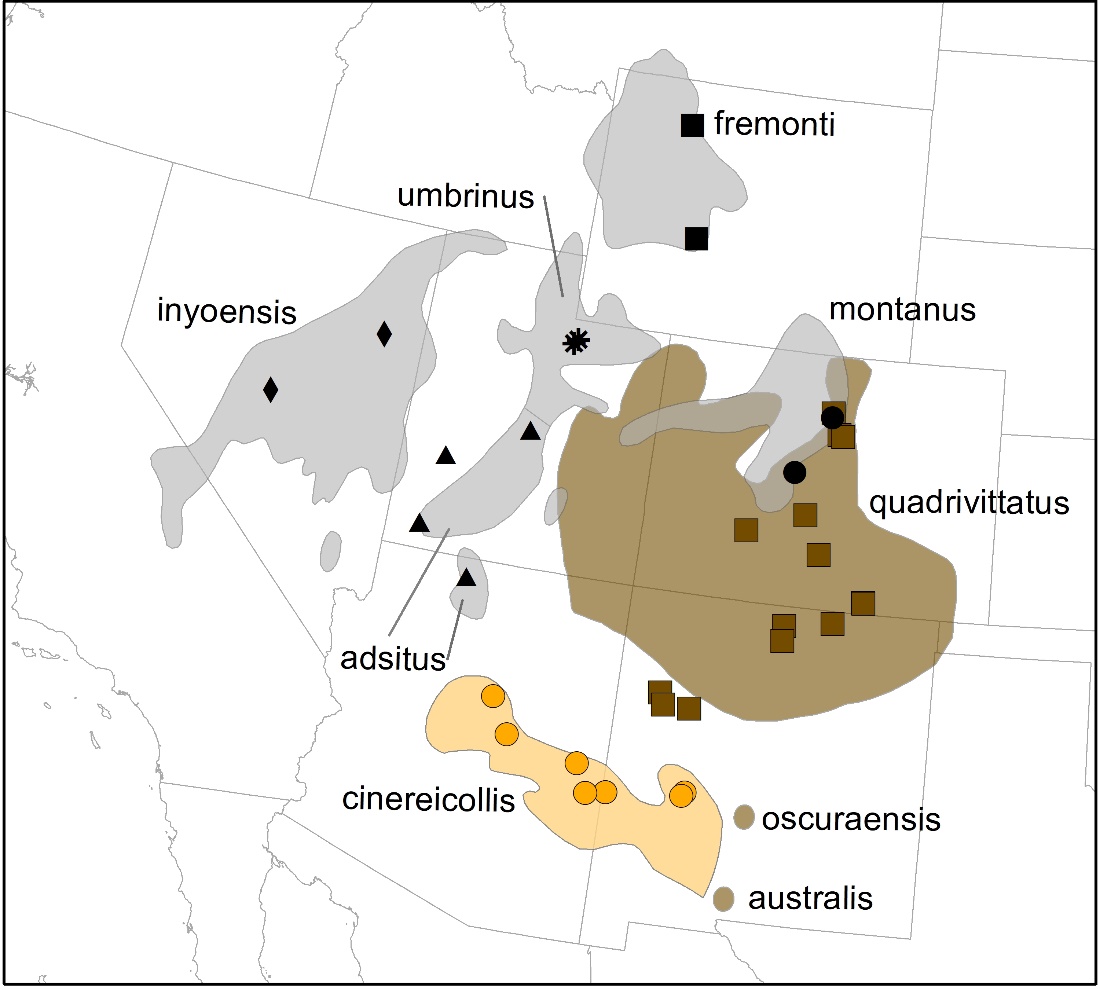


**Figure S1-** Map of outgroup samples from *Neotamias quadrivittatus* (brown; squares), *N. cinereicollis* (gold; circles), and *N. umbrinus* (black; symbology similar to Figure 4 based on museum subspecies designations) with subspecies ranges denoted next to polygons (except for *N. u. nevadensis* and *N. u. sedulus*). The *Tamias striatus* sample was from New Hampshire, USA (not shown). Genetic data and locations for samples shown was obtained from NCBI SRA SRP091305 (Sarver et al., 2017).

**
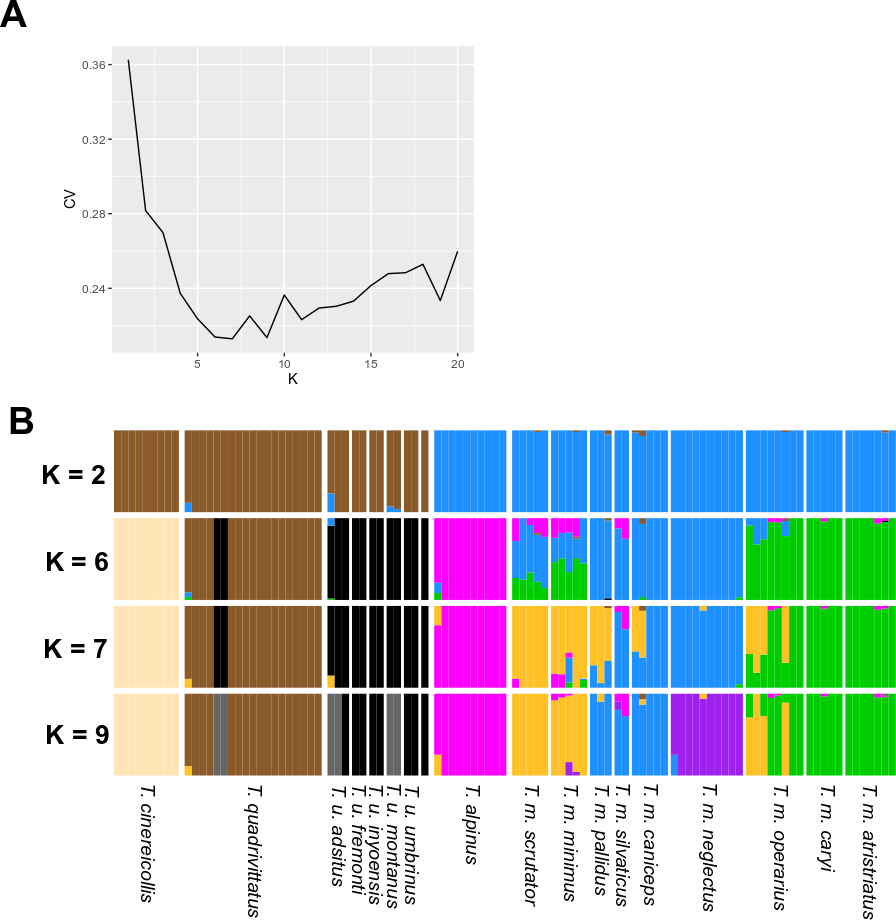
**

**Figure S2-** Ancestry of five *Neotamias* species was inferred using ADMIXTURE. (A) Cross-validation error (CV) plot for 100 samples with a dataset of 513 SNPs. Clustering from the full dataset was run from 1 to 20 clusters (K); at each cluster 20 repetitions of the program were run to estimate the CV error. (B) Ancestry plots for clusters with low CV error, where K = 7 was best supported by the data.

**Figure S3-** Marginal distribution of model parameter beta, the estimated effect of time on diversity.  The 95% credible interval does not include 0, indicating a significant effect of time on individual heterozygosity.

**Table S1-** Samples used in study with location, sampling year, and NCBI SRA Accession number for raw genomic reads. For samples without specific coordinates, we denoted coordinates close to the museum records for plotting on maps. Museum acronyms in Sample ID column include: ANSP: Academy of Natural Sciences of Drexel University; AMNH: American Museum of Natural History; DMNS: Denver Museum of Nature & Science; MVZ: Museum of Vertebrate Zoology at Berkeley University; UMMZ: University of Michigan Museum of Zoology; and UCM: University of Colorado’s Museum of Natural History.

|  |  |  |  |  |  |  |  |
| --- | --- | --- | --- | --- | --- | --- | --- |
| **SampleID** | **Species** | **Subspecies** | **Year** | **Longitude** | **Latitude** | **Sex** | **SRA Accession** |
| AMNH-36856 | alpinus |  | 1903 | -118.25 | 36.83 |  | SAMN19549361 |
| MVZ-207200 | alpinus |  | 2004 | -119.35 | 37.79 |  | [SRR3172049](https://trace.ncbi.nlm.nih.gov/Traces/sra/?run=SRR3172049) |
| MVZ-207201 | alpinus |  | 2004 | -119.34 | 37.80 |  | [SRR3172051](https://trace.ncbi.nlm.nih.gov/Traces/sra/?run=SRR3172051) |
| MVZ-207202 | alpinus |  | 2004 | -119.34 | 37.80 |  | [SRR3172052](https://trace.ncbi.nlm.nih.gov/Traces/sra/?run=SRR3172052) |
| MVZ-207203 | alpinus |  | 2004 | -119.33 | 37.80 |  | [SRR3172053](https://trace.ncbi.nlm.nih.gov/Traces/sra/?run=SRR3172053) |
| MVZ-207204 | alpinus |  | 2004 | -119.33 | 37.80 |  | [SRR3172054](https://trace.ncbi.nlm.nih.gov/Traces/sra/?run=SRR3172054) |
| MVZ-207208 | alpinus |  | 2004 | -119.35 | 37.79 |  | [SRR3172055](https://trace.ncbi.nlm.nih.gov/Traces/sra/?run=SRR3172055) |
| MVZ-207210 | alpinus |  | 2004 | -119.35 | 37.79 |  | [SRR3172056](https://trace.ncbi.nlm.nih.gov/Traces/sra/?run=SRR3172056) |
| MVZ-207212 | alpinus |  | 2004 | -119.35 | 37.79 |  | [SRR3172057](https://trace.ncbi.nlm.nih.gov/Traces/sra/?run=SRR3172057) |
| MVZ-216270 | alpinus |  | 2005 | -119.26 | 37.77 |  | [SRR3172059](https://trace.ncbi.nlm.nih.gov/Traces/sra/?run=SRR3172059) |
| MVZ-217178 | alpinus |  | 2006 | -119.26 | 37.77 |  | [SRR3172060](https://trace.ncbi.nlm.nih.gov/Traces/sra/?run=SRR3172060) |
| DMNS-11378 | cinereicollis |  | 2007 | -111.36 | 34.93 |  | SRR4407904 |
| DMNS-11108 | cinereicollis |  | 2007 | -108.98 | 33.73 |  | SRR4407903 |
| DMNS-11116 | cinereicollis |  | 2007 | -110.98 | 34.38 |  | SRR4407901 |
| DMNS-11115 | cinereicollis |  | 2007 | -109.59 | 34.11 |  | SRR4407900 |
| DMNS-11113 | cinereicollis |  | 2007 | -109.35 | 33.67 |  | SRR4407899 |
| DMNS-11110 | cinereicollis |  | 2007 | -108.98 | 33.73 |  | SRR4407897 |
| DMNS-11111 | cinereicollis |  | 2007 | -109.35 | 33.67 |  | SRR4407898 |
| DMNS-11086 | cinereicollis |  | 2007 | -107.51 | 33.90 |  | SRR4407895 |
| DMNS-11087 | cinereicollis |  | 2007 | -107.56 | 33.84 |  | SRR4407896 |
| ANSP-14651 | minimus | atristriatus | 1931 | -106.01 | 33.09 | female | SAMN19549371 |
| ANSP-14654 | minimus | atristriatus | 1931 | -105.90 | 33.02 | male | SAMN19549372 |
| ANSP-15564 | minimus | atristriatus | 1932 | -105.75 | 33.13 | female | SAMN19549373 |
| ANSP-15566 | minimus | atristriatus | 1932 | -105.99 | 33.08 | male | SAMN19549374 |
| ANSP-15570 | minimus | atristriatus | 1932 | -105.77 | 33.15 | female | SAMN19549375 |
| ANSP-15571 | minimus | atristriatus | 1932 | -105.77 | 33.15 | male | SAMN19549376 |
| ANSP-15579 | minimus | atristriatus | 1932 | -105.32 | 32.95 | male | SAMN19549377 |
| AMNH-35139 | minimus | borealis | 1907 | -116.08 | 51.49 |  | SAMN19549359 |
| AMNH-35140 | minimus | borealis | 1907 | -116.08 | 51.49 |  | SAMN19549360 |
| AMNH-20650 | minimus | caniceps | 1902 | -131.16 | 57.90 |  | SAMN19549349 |
| AMNH-20653 | minimus | caniceps | 1902 | -131.16 | 57.90 |  | SAMN19549350 |
| AMNH-20656 | minimus | caniceps | 1902 | -131.16 | 57.90 |  | SAMN19549351 |
| ANSP-20495 | minimus | caniceps | 1939 | -126.63 | 55.95 | male | SAMN19549380 |
| ANSP-20496 | minimus | caniceps | 1939 | -126.63 | 55.95 | female | SAMN19549381 |
| ANSP-20497 | minimus | caniceps | 1938 | -126.63 | 55.95 | male | SAMN19549382 |
| UCM-8654 | minimus | caryi | 1909 | -105.73 | 37.67 | female | SAMN19549396 |
| UCM-8657 | minimus | caryi | 1909 | -105.59 | 37.66 | female | SAMN19549397 |
| UCM-8660 | minimus | caryi | 1909 | -105.59 | 37.66 | male | SAMN19549398 |
| UCM-8665 | minimus | caryi | 1909 | -105.59 | 37.66 | male | SAMN19549399 |
| UCM-8666 | minimus | caryi | 1909 | -105.59 | 37.66 | female | SAMN19549400 |
| AMNH-140127 | minimus | consobrinus | 1904 | -112.64 | 38.28 |  | SAMN19549348 |
| AMNH-28914 | minimus | consobrinus | 1907 | -106.10 | 40.08 |  | SAMN19549352 |
| AMNH-28915 | minimus | consobrinus | 1907 | -106.10 | 40.08 |  | SAMN19549353 |
| AMNH-39803 | minimus | consobrinus | 1907 | -106.47 | 39.90 |  | SAMN19549366 |
| ANSP-19891 | minimus | jacksoni | 1938 | -89.16 | 45.79 | female | SAMN19549378 |
| ANSP-19895 | minimus | jacksoni | 1938 | -89.16 | 45.79 | male | SAMN19549379 |
| UCM-5632 | minimus | minimus | 1952 | -108.13 | 40.69 | male | SAMN19549389 |
| UCM-5721 | minimus | minimus | 1952 | -107.88 | 40.53 | female | SAMN19549390 |
| UCM-5762 | minimus | minimus | 1952 | -108.82 | 40.92 | male | SAMN19549393 |
| UCM-8642 | minimus | minimus | 1907 | -107.88 | 40.53 | female | SAMN19549394 |
| UCM-8644 | minimus | minimus | 1907 | -108.15 | 40.63 | male | SAMN19549395 |
| ANSP-13306 | minimus | neglectus | 1925 | -94.67 | 49.98 | male | SAMN19549370 |
| UCM-8761 | minimus | neglectus | 1906 | -89.42 | 45.63 | male | SAMN19549401 |
| UCM-8762 | minimus | neglectus | 1906 | -89.32 | 45.66 | male | SAMN19549402 |
| UCM-8765 | minimus | neglectus | 1909 | -89.17 | 45.50 | female | SAMN19549403 |
| UMMZ-32224 | minimus | neglectus | 1904 | -89.75 | 46.78 |  | SAMN19549413 |
| UMMZ-32225 | minimus | neglectus | 1904 | -89.75 | 46.78 |  | SAMN19549414 |
| UMMZ-40253 | minimus | neglectus | 1909 | -87.79 | 45.92 |  | SAMN19549415 |
| UMMZ-48677 | minimus | neglectus | 1916 | -86.49 | 46.43 |  | SAMN19549416 |
| UMMZ-48678 | minimus | neglectus | 1916 | -86.49 | 46.43 |  | SAMN19549417 |
| UMMZ-48679 | minimus | neglectus | 1916 | -86.49 | 46.43 |  | SAMN19549418 |
| AMNH-28942 | minimus | operarius | 1908 | -104.77 | 38.88 |  | SAMN19549354 |
| AMNH-28943 | minimus | operarius | 1907 | -105.97 | 39.41 |  | SAMN19549355 |
| UCM-11793 | minimus | operarius | 1909 | -104.93 | 38.86 | female | SAMN19549385 |
| UCM-18924 | minimus | operarius | 2009 | -105.48 | 38.13 | female | SAMN19549386 |
| UCM-18928 | minimus | operarius | 2009 | -105.48 | 38.13 | female | SAMN19549387 |
| UCM-8776 | minimus | operarius | 1906 | -104.88 | 38.83 | male | SAMN19549404 |
| UCM-8815 | minimus | operarius | 1909 | -105.33 | 38.12 | male | SAMN19549405 |
| UCM-8816 | minimus | operarius | 1909 | -105.33 | 38.12 | male | SAMN19549406 |
| AMNH-41425 | minimus | pallidus | 1917 | -113.46 | 48.23 |  | SAMN19549367 |
| AMNH-41428 | minimus | pallidus | 1917 | -113.46 | 48.23 |  | SAMN19549368 |
| AMNH-41429 | minimus | pallidus | 1917 | -113.46 | 48.23 |  | SAMN19549369 |
| AMNH-33397 | minimus | scrutator | 1912 | -117.94 | 44.32 |  | SAMN19549356 |
| AMNH-33407 | minimus | scrutator | 1912 | -117.94 | 44.32 |  | SAMN19549357 |
| AMNH-33413 | minimus | scrutator | 1912 | -117.94 | 44.32 |  | SAMN19549358 |
| AMNH-39761 | minimus | scrutator | 1908 | -117.82 | 44.77 |  | SAMN19549362 |
| AMNH-39787 | minimus | scrutator | 1908 | -117.67 | 44.77 |  | SAMN19549363 |
| AMNH-39794 | minimus | scrutator | 1908 | -117.24 | 43.98 |  | SAMN19549364 |
| AMNH-39795 | minimus | scrutator | 1908 | -117.24 | 43.98 |  | SAMN19549365 |
| ANSP-21629 | minimus | silvaticus | 1984 | -103.78 | 44.34 | male | SAMN19549383 |
| ANSP-21630 | minimus | silvaticus | 1984 | -103.78 | 44.34 | male | SAMN19549384 |
| AMNH-132728 | quadrivittatus | quadrivittatus | 1938 | -108.22 | 35.40 |  | SAMN19549344 |
| AMNH-132729 | quadrivittatus | quadrivittatus | 1938 | -108.22 | 35.40 |  | SAMN19549345 |
| AMNH-132730 | quadrivittatus | quadrivittatus | 1938 | -108.22 | 35.40 |  | SAMN19549346 |
| AMNH-132731 | quadrivittatus | quadrivittatus | 1938 | -108.22 | 35.40 |  | SAMN19549347 |
| UCM-5497 | quadrivittatus | quadrivittatus | 1952 | -106.97 | 38.10 | female | SAMN19549388 |
| UCM-5747 | quadrivittatus | quadrivittatus | 1952 | -105.50 | 40.09 | female | SAMN19549391 |
| UCM-5750 | quadrivittatus | quadrivittatus | 1952 | -105.50 | 40.09 | female | SAMN19549392 |
| UCM-8891 | quadrivittatus | quadrivittatus | 1909 | -105.84 | 38.45 | female | SAMN19549407 |
| UCM-8892 | quadrivittatus | quadrivittatus | 1909 | -105.84 | 38.45 | female | SAMN19549408 |
| UCM-8900 | quadrivittatus | quadrivittatus | 1909 | -105.49 | 37.85 | female | SAMN19549409 |
| UCM-8907 | quadrivittatus | quadrivittatus | 1909 | -104.52 | 37.17 | female | SAMN19549410 |
| UCM-8908 | quadrivittatus | quadrivittatus | 1909 | -104.52 | 37.17 | female | SAMN19549411 |
| UCM-8958 | quadrivittatus | quadrivittatus | 1909 | -104.51 | 37.16 | male | SAMN19549412 |
| DMNS-11136 | quadrivittatus | quadrivittatus | 2007 | -107.63 | 35.21 |  | SRR4407894 |
| DMNS-11814 | quadrivittatus | quadrivittatus | 2008 | -105.07 | 36.80 |  | SRR4407893 |
| DMNS-11031 | quadrivittatus | quadrivittatus | 2007 | -105.33 | 39.76 |  | SRR4407883 |
| DMNS-11024 | quadrivittatus | quadrivittatus | 2007 | -105.25 | 39.74 |  | SRR4407884 |
| DMNS-11078 | quadrivittatus | quadrivittatus | 2007 | -106.01 | 36.68 |  | SRR4407885 |
| DMNS-11085 | quadrivittatus | quadrivittatus | 2007 | -106.01 | 36.44 |  | SRR4407886 |
| DMNS-11134 | quadrivittatus | quadrivittatus | 2007 | -107.63 | 35.21 |  | SRR4407888 |
| DMNS-11818 | quadrivittatus | quadrivittatus | 2008 | -108.13 | 35.22 |  | SRR4407889 |
| Tstri.#2 | striatus | lysteri | 2010 | -71.03 | 43.82 |  | SRR504642 |
| AMNH-238173 | umbrinus | adsitus | 1917 | -113.39 | 37.40 |  | SAMN19974811 |
| DMNS-11625 | umbrinus | montanus | 2008 | -106.15 | 39.10 |  | SRR4407872 |
| DMNS-11379 | umbrinus | adsitus | 2007 | -112.28 | 36.70 |  | SRR4407873 |
| DMNS-11160 | umbrinus | umbrinus | 2007 | -110.99 | 40.61 |  | SRR4407874 |
| DMNS-11165 | umbrinus | umbrinus | 2007 | -110.91 | 40.68 |  | SRR4407875 |
| DMNS-11147 | umbrinus | fremonti | 2007 | -109.25 | 44.30 |  | SRR4407876 |
| DMNS-11681 | umbrinus | inyoensis | 2008 | -116.86 | 38.92 |  | SRR4407877 |
| DMNS-11700 | umbrinus | unlisted-utah | 2008 | -111.56 | 39.15 |  | SRR4407878 |
| DMNS-11687 | umbrinus | inyoensis | 2008 | -114.86 | 40.18 |  | SRR4407879 |
| DMNS-11881 | umbrinus | fremonti | 2008 | -108.80 | 42.54 |  | SRR4407881 |
| DMNS-11433 | umbrinus | montanus | 2008 | -105.51 | 40.02 |  | SRR4407882 |

**Table S2-** Pairwise *F_ST_* based on 513 SNPs of *Neotamias* (sub)species (Figures 1 and S1 for geographic locations) with significant values identified in bold.

|  | *N. cinereicollis* | *N. quadrivittatus* | *N. u. umbrinus* | *N. u. inyoensis* | *N. u. fremonti* | *N. u. adsitus* | *N. u. montanus* | *N. alpinus* | *N. m. scrutator* | *N. m. minimus* | *N. m. silvaticus* | *N. m. pallidus* | *N. m. caniceps* | *N. m. neglectus* | *N. m. consobrinus^1^* | *N. m. operarius* | *N. m. caryi* | *N. m. atristriatus* |
| --- | --- | --- | --- | --- | --- | --- | --- | --- | --- | --- | --- | --- | --- | --- | --- | --- | --- | --- |
| *N. cinereicollis* | - |  |  |  |  |  |  |  |  |  |  |  |  |  |  |  |  |  |
| *N. quadrivittatus* | **0.339** | - |  |  |  |  |  |  |  |  |  |  |  |  |  |  |  |  |
| *N. u. umbrinus* | **0.658** | **0.506** | - |  |  |  |  |  |  |  |  |  |  |  |  |  |  |  |
| *N. u. inyoensis* | 0.661 | **0.500** | -0.027 | - |  |  |  |  |  |  |  |  |  |  |  |  |  |  |
| *N. u. fremonti* | **0.662** | **0.511** | -0.100 | 0.057 | - |  |  |  |  |  |  |  |  |  |  |  |  |  |
| *N. u. adsitus* | **0.632** | **0.483** | 0.040 | -0.021 | 0.080 | - |  |  |  |  |  |  |  |  |  |  |  |  |
| *N. u. montanus* | **0.664** | **0.497** | 0.114 | 0.070 | 0.186 | 0.122 | - |  |  |  |  |  |  |  |  |  |  |  |
| *N. alpinus* | **0.675** | **0.568** | **0.665** | **0.657** | **0.668** | **0.603** | **0.656** | - |  |  |  |  |  |  |  |  |  |  |
| *N. m. scrutator* | **0.622** | **0.493** | 0.558 | **0.542** | 0.554 | **0.457** | **0.551** | **0.303** | - |  |  |  |  |  |  |  |  |  |
| *N. m. minimus* | **0.610** | **0.485** | **0.562** | **0.560** | 0.560 | **0.485** | **0.553** | **0.265** | **0.127** | - |  |  |  |  |  |  |  |  |
| *N. m. silvaticus* | **0.672** | **0.546** | 0.678 | 0.678 | 0.674 | 0.548 | 0.700 | **0.368** | **0.250** | 0.099 | - |  |  |  |  |  |  |  |
| *N. m. pallidus* | **0.662** | **0.556** | 0.605 | 0.596 | 0.602 | 0.513 | 0.611 | **0.446** | **0.207** | **0.223** | 0.244 | - |  |  |  |  |  |  |
| *N. m. caniceps* | **0.664** | **0.561** | **0.623** | 0.613 | **0.617** | **0.546** | 0.624 | **0.437** | **0.275** | **0.226** | 0.159 | 0.137 | - |  |  |  |  |  |
| *N. m. neglectus* | **0.668** | **0.571** | **0.656** | **0.648** | **0.656** | **0.587** | **0.649** | **0.431** | **0.291** | **0.242** | **0.291** | **0.343** | **0.312** | - |  |  |  |  |
| *N. m. consobrinus^1^* | **0.624** | **0.506** | **0.569** | 0.556 | 0.562 | **0.482** | 0.553 | **0.386** | **0.129** | **0.163** | 0.289 | **0.257** | **0.310** | **0.348** | - |  |  |  |
| *N. m. operarius* | **0.661** | **0.543** | 0.596 | 0.589 | **0.591** | 0.515 | **0.582** | **0.424** | **0.249** | **0.223** | 0.344 | **0.344** | **0.372** | **0.424** | **0.157** | - |  |  |
| *N. m. caryi* | **0.646** | **0.532** | **0.568** | **0.559** | 0.567 | **0.495** | **0.547** | **0.407** | **0.223** | **0.215** | 0.345 | **0.336** | **0.376** | **0.400** | **0.159** | -0.004 | - |  |
| *N. m. atristriatus* | **0.649** | **0.535** | **0.598** | **0.587** | **0.593** | **0.520** | **0.579** | **0.391** | **0.206** | **0.246** | **0.385** | **0.351** | **0.388** | **0.426** | **0.161** | **0.094** | **0.128** | - |
| ^1^- These samples were classified in the museum as *N. m. operarius*. | | | | | | | | |  |  |  |  |  |  |  |  |  |  |

**Text S1**- Supplemental methods for subsetting *Neotamias* probe set.

We genotyped samples using a reduced version of an exon probe set previously designed for *Neotamias* (Bi et al., 2013; Bi et al., 2012). The original probe set was designed using transcriptomic data from *N. alpinus* and validated in *N. ruficaudus*, *N. ameonus*, and *Tamias striatus* (Bi et al., 2012). To determine which exons to include in our sequencing, we analyzed levels of polymorphism using existing *Neotamias* sequence data generated from the full probe seN. Specifically, we downloaded 11 *N. alpinus* (SRA accessions: SRR 31719[70, 71, 99], 31720[11, 12, 22, 36, 38, 50, 61, 62]) and 11 *N. quadrivittatus* (SRA accessions: SRR 44078[83-90, 92-94]; Table S1) datasets, and aligned the reads against a FASTA file containing the probes using BOWTIE2 v2.3.1 with default settings (Langmead & Salzberg, 2012). We called SNPs from all 22 samples combined by first running SAMTOOLS v1.3.1 mpileup (Li et al., 2009), then BCFTOOLS v1.3.1 with the consensus caller (-c) and allowed for indels. We ran a Fisher’s exact test in PLINK v1.9 (Chang et al., 2015; Purcell et al., 2007) to compare allele frequencies between the two species. We summarized data for each exon by counting the number of SNPs and averaging the minor allele frequency (MAF) across SNPs within a contig for each species. We then assigned each exon to one of the following categories: mapped, mapped with low minor allele frequency (MAF; where one species was polymorphic but the other was not), unmapped, mitochondria, and X or Y chromosome. Using the gene model annotations (Bi et al., 2012), we removed contigs associated with the X and Y chromosomes.

Of the 22,838 exons in the full probe set, 18,566 mapped to the 22 samples from the SRA. To thin the probe set, we first removed contigs outside of the range of 500 - 5,000 bp, then estimated number of variable sites (1-15 bp) and MAF for each contig per species. We selected 3,617 exons from the mapped reads capturing a range of MAF from each species, 25 exons where no data mapped, and 27 exons where MAF was 0 in one species but >0 in the other. Arbor BioSciences (Ann Arbor, MI) designed a 50% overlapping tile deign with 100 nucleotide long probes, filtered for specificity in rodents, for a total of 57,509 baits to sequence 4Mb of the nuclear genome. The mitochondrial genome was captured using a separate 196 bait panel of probes designed using the *N. quadrivittatus* mitochondrial genome (NCBI accession number KY070142).

**Text S2-** R script used to remove C to T and G to A transitions, then select a single locus within each contig with the lowest missing data. Assumes PLINK input files from --make-bed --missing and --freq, and writes an output file for use with PLINK --extract flag.

library(data.table)

bim <- read.table("Neotamias.bim",header=F)

miss <- read.table("Neotamias.lmiss",header=T)

freq <- read.table("Neotamias.frq",header=T)

d <- cbind(bim,miss,freq)

#Remove sites with missing data called by ANGSD

d1 = subset(d, !(V5 == 0))

#remove aDNA risky alleles

d2 <- subset(d1, !(V5 == "C" & V6 == "T"))

d3 <- subset(d2, !(V5 == "G" & V6 == "A"))

#output data

d3 <- as.data.table(d3)

out <- d3[d3[, .I[which.min(F_MISS)], by=CHR]$V1]

write.table(out[,2],"Extract.txt",sep="\t",quote=F,col.names=F,row.names=F)

**Text S3**- Alternative model of heterozygosity over time using a Poisson regression.

To account for possible model mis-specification due to both the heteroscedasticity in the data and the fact that our response variable (heterozygosity) is constrained to vary between 0 and 1, while the distribution we used to model it (the multivariate normal) was not, we also analyzed the data using a Poisson regression approach. In this approach, we modeled the number of heterozygous sites in each individual as a draw from a Poisson distribution. To accommodate heterogeneous exposure due to differences in the number of genotyped base-pairs between individuals, we included an offset term for each sample equal to the log of the number of genotyped base-pairs in that sample. As with the multivariate normal (MVN) model presented in the main text, we wished to control for spatial autocorrelation in the data. To incorporate spatial structure, we nested the MVN model within the Poisson regression, as shown below:

$$X_{i} \sim Poisson({}_{i}= \exp({}_{i} + \log(n_{i})))$$

$${}_{i} \sim MVN \left( \mu_{i}= M+ \beta{\times T}_{i}, \Sigma= \alpha_{0}\times exp\left( -\left( \alpha_{1}D \right)^{\alpha_{2}} \right) \right)$$

where *X_i_* is the number of heterozygous sites in the *i*^th^ individual, *n_i_* is the number of genotyped base-pairs in the *i*^th^ individual, $\mu_{i}$ is the log expected value of the number of heterozygous sites in the *i*^th^ individual (ignoring the offset), *M* is the global intercept, $\beta$ is the estimated per-year effect of time on heterozygosity, $T_{i}$ is the sampling year of the *i*^th^ individual, *D* is the pairwise geographic distance between all individuals, and the $\alpha$ parameters govern the shape of the decay of covariance in heterozygosity between individuals with geographic distance. As in the MVN model, the priors on all parameters except for *α_2_* were standard normals (N(0,1)). The prior on *α_2_* was uniform between 0 and 2 (U(0,2)), which are the parameter limits over which the powered exponential function is stable. The model was implemented in RStan v2.21.2 (Stan Development Team, 2020).

The mean estimated marginal posterior distribution on $\beta$ (the log of the estimated per-year effect of time on heterozygosity) was −4.94 × 10^-3^ (95% credible interval: −5.22 × 10^-3^ to −4.66 × 10^-3^), indicating a statistically significant estimated decline in heterozygosity through time. Note that, in the Poisson regression, $\beta$ is the per-year effect on the expected number of heterozygous sites in an individual, and not (as in the MVN-model) the per-year effect on the expected heterozygosity. The posterior predictive checks (shown below as Figure T3) demonstrated good model fit.

**
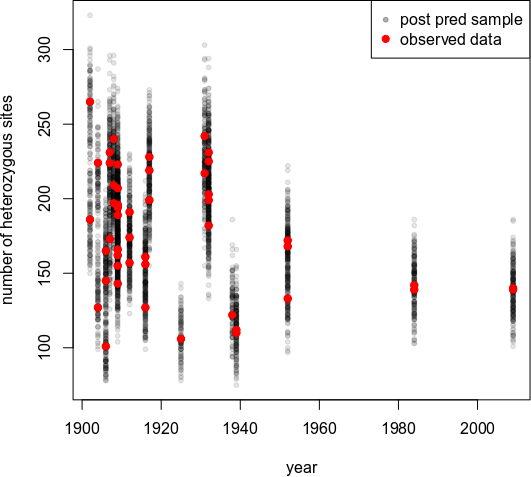
**

**Figure T3-** Posterior predictive check on Poisson regression model. Red points are observed data, gray points are posterior predictive draws.

**LITERATURE CITED**

Bi, K., Linderoth, T., Vanderpool, D., Good, J. M., Nielsen, R. & Moritz, C. 2013. Unlocking the vault: next-generation museum population genomics. *Molecular Ecology* **22**: 6018-6032.

Bi, K., Vanderpool, D., Singhal, S., Linderoth, T., Moritz, C. & Good, J. M. 2012. Transcriptome-based exon capture enables highly cost-effective comparative genomic data collection at moderate evolutionary scales. *BMC Genomics* **13**: 403.

Chang, C. C., Chow, C. C., Tellier, L. C. A. M., Vattikuti, S., Purcell, S. M. & Lee, J. J. 2015. Second-generation PLINK: rising to the challenge of larger and richer datasets. *GigaScience* **4**: 7.

Langmead, B. & Salzberg, S. L. 2012. Fast gapped-read alignment with Bowtie 2. *Nature Methods* **9**: 357-359.

Li, H., Handsaker, B., Wysoker, A., Fennell, T., Ruan, J., Homer, N., Marth, G., Abecasis, G. & Durbin, R. 2009. The Sequence Alignment/Map format and SAMtools. *Bioinformatics* **25**: 2078-9.

Purcell, S., Neale, B., Todd-Brown, K., Thomas, L., Ferreira, M. A. R., Bender, D., Maller, J., Sklar, P., de Bakker, P. I. W., Daly, M. J. & Sham, P. C. 2007. PLINK: A tool set for whole-genome association and population-based linkage analyses. *The American Journal of Human Genetics* **81**: 559-575.

Sarver, B. A. J., Demboski, J. R., Good, J. M., Forshee, N., Hunter, S. S. & Sullivan, J. 2017. Comparative Phylogenomic Assessment of Mitochondrial Introgression among Several Species of Chipmunks (*Tamias*). *Genome Biology and Evolution* **9**: 7-19.

Stan Development Team (2020) RStan: the R interface to Stan. . pp., http://mc-stan.org/.
